# Demonstration and Characterization of Cyst-Like Structures in the Life Cycle of *Trichomonas vaginalis*

**DOI:** 10.3389/fcimb.2019.00430

**Published:** 2020-01-14

**Authors:** Divya Beri, Priya Yadav, H. R. Nandini Devi, Chinmaya Narayana, Darshak Gadara, Utpal Tatu

**Affiliations:** ^1^Department of Biochemistry, Indian Institute of Science, Bangalore, India; ^2^Indira Clinic, Bangalore, India

**Keywords:** *Trichomonas*, cyst-like structures, encystation, excystation, proteomics

## Abstract

*Trichomonas vaginalis* is the parasitic protozoan residing in human urogenital tract causing trichomoniasis, which is the leading non-viral sexually transmitted disease. It has cosmopolitan distribution throughout the globe and affects both men and women. Lifecycle of the parasite has been traditionally described as consisting of motile and symptom-causing trophozoites. Chemical and temperature perturbations in trophozoites have been shown to aid conversion to pseudocysts, which is poorly investigated. In the current study, we show the formation of viable cyst-like structures (CLS) in stationary phase of *T. vaginalis* axenic culture. We used a fluorescent stain called calcofluor white, which specifically binds to chitin and cellulose-containing structures, to score for *T. vaginalis* CLS. Using flow cytometry, we demonstrated and quantitated the processes of encystation as well as excystation; thus, completing the parasite's lifecycle *in vitro* without any chemical/temperature alterations. Like cysts from other protozoan parasites such as *Entamoeba histolytica* and *Giardia lamblia, T. vaginalis* CLS appeared spherical, immotile, and resistant to osmotic lysis and detergent treatments. Ultrastructure of CLS demonstrated by Transmission Electron Microscopy showed a thick electron-dense deposition along its outer membrane. To probe the physiological role of CLS, we exposed parasites to vaginal pH and observed that trophozoites took this as a cue to convert to CLS. Further, upon co- culturing with cells of cervical origin, CLS rapidly excysted to form trophozoites which abrogated the cervical cell monolayer in a dose-dependent manner. To further corroborate the presence of two distinct forms in *T. vaginalis*, we performed two-dimensional gel electrophoresis and global, untargeted mass spectrometry to highlight differences in the proteome with trophozoites. Interestingly, CLS remained viable in chlorinated swimming pool water implicating the possibility of its role as environmentally resistant structures involved in non-sexual mode of parasite transmission. Finally, we showed that symptomatic human patient vaginal swabs had both *T. vaginalis* trophozoites and CLS; thus, highlighting its importance in clinical infections. Overall, our study highlights the plasticity of the pathogen and its rapid adaption when subjected to stressful environmental cues and suggests an important role of CLS in the parasite's life cycle, pathogenesis and transmission.

## Introduction

*Trichomonas vaginalis* is a protozoan parasite and the causative agent of the most common non-viral, sexually transmitted disease (STD) in humans known as trichomoniasis (Schwebke and Burgess, 2004). The World Health Organization (WHO) estimates that more than 270 million people are infected with *T. vaginalis* annually throughout the world (Newman et al., 2015). A recent study reported that 56% patients attending STD clinics were infected with *T. vaginalis* (Johnston and Mabey, 2008). The symptoms of *T. vaginalis* range from being completely asymptomatic to acute inflammation, premature labor and low birth-weight of infants in pregnant women, vaginitis, increased susceptibility to life-threatening Human immunodeficiency Virus (HIV) infection, cervical neoplasia, and pelvic inflammatory disease (Walther, 1973; Johnston and Mabey, 2008). The urogenital tract serves as the physiological niche of the parasite and presents many challenges to the resident parasite. *T. vaginalis* is mostly prevalent in women in their reproductive age. Thus, the parasite must deal with changes in vaginal epithelium, cervical mucus, pH, redox potential, and overall modulation of the vaginal microbiome that occurs during the menstrual cycle (Demes et al., 1988). The survival and success of the parasite requires a robust and specialized machinery to tackle these stresses. The only drug of choice for trichomoniasis is metronidazole and drug-resistance is on the rise (Dunne et al., 2003).

Most studies on *T. vaginalis* have reported only the trophozoite form as the active, motile, and infective form of the parasite which is sexually transmitted between individuals (Warton and Honigberg, 1979) and amoeboid form characterized by an increase in surface contact with epithelial cells from vagina, cervix, and prostate (Hirt, 2013). Recent reports have suggested formation of pseudocysts which are described as structures having internalized flagella in endocytic vesicles where they continue their beating motion (Pereira-Neves et al., 2003), rounded shape, non-motile and lacking a true cyst wall (Dias-Lopes et al., 2017). Pseudocyst was reported to be induced by temperature variations and iron depletion in the media in *in-vitro* culture of *T. vaginalis*. Such structures were also reported to be formed in closely-related *T. foetus* under temperature variation, iron depletion and by chemicals like hydroxyurea (Ribeiro et al., 2002). Previously some researchers believed pseudocysts to be degenerative structures (Petrin et al., 1998) but recent studies have highlighted its role in cervical neoplasia patients and transmission in rats (Zarei et al., 2018). Further, a recent comparative study of proteomes of *T. vaginalis* trophozoites and pseudocysts reported major differences between protein content and abundance of various sets of proteins in the two forms (Dias-Lopes et al., 2018). Recent work in the field has highlighted the relevance of non-sexual modes of transmission of the parasite from contaminated douche nozzle, toilet seats, and swimming pools (Pereira-Neves and Benchimol, 2008). However, there remains a gap in our knowledge regarding how the parasite is non-sexually transmitted. Though, *T. vaginalis* pseudocyst has been defined, its existence, pathology, and role in the parasite's life cycle are poorly studied (Pereira-Neves et al., 2003; Hussein and Atwa, 2008; Afzan and Suresh, 2012; Dias-Lopes et al., 2017).

In the current study we have characterized the cyst like structure (CLS) in *T. vaginalis*. Presence of chitin is a characteristic feature of cysts in other protozoan parasites like *Entamoeba histolytica* and *Giardia lamblia* (Arroyo-Begovich et al., 1980; Ward et al., 1985). Thus, using two fluorescent dyes which uniquely bind to chitin [Calcofluor White; CFW (Kawamoto et al., 1987) and Alexafluor-conjugated Wheat Germ Agglutinin; WGA (Chatterjee et al., 2009)], we have scored for encystation and excystation in axenic *in vitro* culture of lab passaged as well as fresh human clinical isolates of *T. vaginalis*. Thus, without the use of any temperature perturbations and/or chemical agents, we were successful in achieving encystation and excystation of *T. vaginalis in vitro*. The observation agrees well with a previous study which had identified chitin in the cell surface of *T. vaginalis* and *T. foetus* using anti-chitin antibody (Kneipp et al., 1998). Further, we have characterized *T. vaginalis* CLS and in contrast to trophozoites, demonstrated it to be resistant to adverse environmental conditions, namely, water, detergent and acidic vaginal pH treatments. A 2-D gel electrophoresis analysis followed by global mass spectrometry of the two forms showed stark differences between their proteomic profiles. Additionally, transmission electron microscopy of the two forms revealed unique deposition of presumably chitin along the membrane of CLS with dimensions comparable to thickness of cyst wall from related parasites. We thus, propose nomenclature of these forms as CLS, rather than pseudocysts as these forms have hallmarks of true cysts as enlisted above. Finally, we demonstrated the presence of both trophozoite and CLS in human female clinical cases of *T. vaginalis* which strengthens our hypothesis of relevance of cyst-form in *in vivo* disease condition. Together, our results provide credence to the possibility that *T. vaginalis* may not exclusively be an STD and additional level of caution must be exercised by clinicians when diagnosing and treating patients with this disease.

## Materials and Methods

### Parasite Culturing

*Trichomonas vaginalis* isolate-1 (Strain is a kind gift from Prof. Daman Saluja and Dr. Manisha Yadav, ACBR, New Delhi) was cultured in glass tubes in TYI-S-33 medium at 37°C, as detailed before (Singh et al., 2018). *Trichomonas vaginalis* isolate-2 was a fresh clinical isolate cultured from vaginal swab specimen of a symptomatic patient and experiments were performed before it had undergone 5 lab passages. Additionally, the medium contained 10% heat inactivated adult bovine serum (HiMedia), PenStrep (HiMedia), and 2.5% Diamond Vitamin mix (Sigma) with a pH of 6.8 (Singh et al., 2018).

### Encystation Assay

*Trichomonas vaginalis* log-phase trophozoites were inoculated in fresh medium and allowed to grow at 37°C up to 60 h without change of culture medium. Every 12 h, cells were stained with 0.1% Calcofluor White (CFW) and Fluorescein diacetate (FDA) for determining percentage encystation. This was followed by performing flow cytometry at different time points.

Representative microscopic images were captured using a confocal scanning microscope (Leica TCS SP8) using 63X magnification of the objective lens (Supplementary Figure 1).

### Viability Assay

A cell- permeant esterase substrate Fluorescein diacetate (FDA) was used to determine viability of the cyst-like structures during encystation, excystation, incubation with swimming pool water and post detergent treatment. The assay tested the ability of CLS to convert FDA into Fluorescein, a method previously used to determine viability of cysts of *E. histolytica* (Aguilar-Díaz et al., 2010). FDA can enter viable trophozoites and CLS of *T. vaginalis*, as evident from fluorescence microscopic images in Supplementary Figure 4. Cells were adjusted to 1 × 10^6^/ml in 200 μl PBS after 2 washes with 1X PBS. These cells were treated with 3 μl of FDA stock solution (2.5 mg/ml in Acetone; Sigma) and incubated for 10 min in dark at room temperature. Similarly, *T. vaginalis* CLS was stained with Propidium Iodide (PI) at a final concentration of 0.1 mg/ml (stock solution 2 mg/ml in water). PI stains only dead cells while live cells exclude PI. Percentage viability was determined by using flow cytometry.

### Enrichment of Cyst-Like Structures

*Trichomonas vaginalis* log-phase trophozoites were inoculated in fresh medium and allowed to grow at 37°C for 48 h. Following this, cells were pelleted down at 500 g for 5 min and resuspended in sterile double distilled water. The tube was placed in an end-to-end rotor for 12 h at 4°C. Post incubation, CFW staining was performed. As shown in Supplementary Figure 2B, CLS resist incubation in water while trophozoites lysed due to changes in surrounding osmolarity. Thus, this treatment preferentially enriched *T. vaginalis* CLS.

### Wheat Germ Binding Assay

Trophozoites and enriched CLS were harvested and washed in PBS twice and stained with Alexafluor 488-conjugated wheat germ agglutinin at a concentration of 20 μg/ml for 60 min at 4°C and microscopy was performed by using 100X (Chatterjee et al., 2009).

### Excystation Assay

Enriched CLS forms were washed twice in 1X PBS pH 7.4 and inoculated in fresh medium (1x 10^6^ cells/ml). Medium was replenished every 24 h. Cells were checked for viability every 24 h by incubating with FDA and CFW followed by flow cytometry upto 86 h. Also, CFW stained cells were observed using a confocal scanning microscope (Leica TCS SP8) and the representative images were captured (Supplementary Figure 3).

### Osmotic Resistance Assay

2 × 10^6^ enriched CLS (prepared as described above) and log-phase trophozoites were resuspended in sterile water at 37°C. At 0, 2, 4, 8, and 12 h cells were checked for number and viability by using flow cytometry after staining cells with CFW and FDA. The experiments were performed in triplicates. Percentage viable cells was plotted against each time point for both trophozoites and CLS.

For the detergent resistance assay, 2.5 × 10^6^ trophozoites and enriched CLS were suspended in two concentrations of the detergent, sodium dodecyl sulfate (SDS); 0.05 and 0.1%. Cells were pelleted after treatment and stained with CFW and viability assay was performed using FDA. Flow cytometry was used to quantitate viable CLS at 0, 2, 4, and 6 h.

Swimming pool water was collected from Indian Institute of Science, Bangalore containing 1.2 ppm free residual chloride pH 7.2 and filtered with 0.22 μm filter (Millipore) to get rid of any particulate matter. 2 × 10^6^ trophozoites and enriched CLS were resuspended in pool water and incubated at 37°C. The cells were counted, and viability was checked at 0, 2, 4, and 6 h. Percentage viable cells were plotted against time for both trophozoites and CLS again using flow cytometry with CFW staining and FDA viability assay. All experiments were done in triplicates.

### Assay for Encystation of *T. vaginalis* Trophozoites at Different pH

*Trichomonas vaginalis* trophozoites were grown to log phase and harvested. 2 × 10^6^ pure trophozoites were inoculated in TYI medium set to pH 3, pH 5, and pH 6.8 and allowed to grow at 37°C. Post 12, 24, 36, and 48 h, cells were harvested and stained with 0.1% CFW in PBS and incubated for 10 min in dark followed by washing with PBS thrice. One thousand cells were counted to calculate percentage encystation at different pH of TYI medium.

$$\begin{array}{l} \text{Percentage encystation}\\ \;=\;\frac{\text{Number of cells stained with CFW }\left(\text{CLS}\right)}{\text{Total Number of cells}}\times 100 \end{array}$$

### Cytotoxicity Assay

HeLa cells were grown in a 24-well plate in DMEM (Sigma) supplemented with 10% fetal bovine serum. Pure trophozoites and enriched CLS were resuspended in TYI: DMEM medium in a ratio of 1:3 and added to a confluent layer HeLa cells in a ratio of *T. vaginalis*: HeLa cells 2:1, 5:1, 10:1, 20:1, and 50:1. The plate was incubated at 37°C, 5% CO for 4 h. Following this, the plate was chilled in ice to allow for the detachment of *T. vaginalis* trophozoites and CLS. 3- [4,5-dimethylthiazol-2-yl]-2,5 diphenyl tetrazolium bromide (MTT) assay was performed, as described elsewhere (Van Meerloo et al., 2011). Absorbance was measured using a spectrophotometer and percentage viability of HeLa cells was calculated and plotted against varying concentrations of *T. vaginalis* trophozoites and CLS.

### Protein Extraction and 2-D Gel Electrophoresis

*Trichomonas vaginalis* trophozoite and enriched CLS were harvested by centrifugation at 500 g for 5 min and washed three times in 1X PBS. The cells were lysed in lysis buffer containing 8 M urea, 4% 3-[(3-cholamidopropyl) dimethylammonio]-1-propanesulfonate CHAPS and 20 mM Tris-Cl pH 7.5 in presence of protease inhibitors (Roche Diagnostics GmbH) followed by 3 freeze- thaw cycles in liquid nitrogen. The whole cell lysates were centrifuged at 12,000 rpm, 4°C for 15 min to remove cell debris. Protein concentration was determined using Bradford assay. The 2D cleanup kit (GE Healthcare) was used to remove impurities from the whole cell lysates. Four hundred micrograms of total protein was diluted to a final volume of 200 μl of rehydration buffer (containing 7M urea, 2M thiourea, 2% CHAPS, 2% IPG Buffer, DTT and 0.5% bromophenol blue) for isoelectric focusing. The samples were loaded onto 11 cm immobilized pH gradient (IPG) gel strips of pH range 4–7 linear gradient by in-gel sample rehydration for 10 h. Isoelectric focusing was performed in a gradient manner in an Ettan IPGphor 3 (GE Healthcare) by using the following settings: 100 V for 2 h; 500 V for 3 h; 1,000 V for 5 h; 3,000 V for 3 h; 5,000 V for 9,000 VH; 5,000 V for 3 h; 500 V for 2 h. After isoelectric focusing, the strips were incubated in equilibration buffer 1 (50 mM Tris-Cl, pH 8.8, 6 M urea, 30% glycerol, 2% SDS, 0.5% Bromophenol Blue, and 130 mM DTT) for 20 min and then in equilibration buffer 2 (50 mM Tris-Cl pH 8.8, 6 M urea, 30% glycerol, 2% SDS, 0.5% Bromophenol Blue and 135 mM Iodoacetamide) for 20 min. Equilibrated IPG strips were placed on top of a 12% polyacrylamide gel and sealed with 1% agarose containing bromophenol blue for second dimension. The gels were run at 35 mA at room temperature.

### Mass Spectrometry Analysis

*Trichomonas vaginalis* pure trophozoites and enriched CLS were lysed as described earlier (Singh et al., 2018). Protein was estimated, and 350 μg protein was precipitated using chilled acetone and incubated overnight at −80°C. Following two acetone washes, the protein pellet was solubilized in 50 mM ammonium bicarbonate pH 8.0. This was followed by treatment with 10 mM DTT at 56°C for 45 min and 20 mM iodoacetamide at room temperature for 1 h. Mass spectrometry grade Gold-trypsin was added (1:50) and incubated at 37°C for 16 h. Following this, the solution was evaporated using a Speed Vac (Eppendorf) and was resolubilized in 0.1% formic acid, 3% acetonitrile in mass spectrometry grade water. Agilent Technologies 6545XT AdvanceBio LC/Q-TOF was used for protein detection and identification. Mobile phase A was 0.1% formic acid in water. Mobile phase B was 0.1% formic acid in Acetonitrile. LC gradient ran details have been provided in Supplementary Methods. Samples were loaded directly into a Agilent AdvanceBio Peptide Map 2.1 × 150 mm, 2.7 μm with an injection volume of 20 μl at a flow rate of 0.400 ml/min./ LC was interfaced to a Q-TOF mass spectrometer (Agilent 6545XT AdvanceBio LC/Q-TOF) via an Electrospray ionization (Dual AJS ESI). The mass spectrometer was programmed to Auto MS2 mode with ions in full scan from 300 to 1,700 m/z value. Isolation width was set to 1.3 Da, with a MS/MS scan rate of 3 spectra/ sec. Proteome Discoverer^TM^ (Thermo Fisher) was used to analyze the proteins and PANTHER and Argot2 (Version 14.0 released 2018-12-03) (Huang Da et al., 2009; Mi et al., 2013; Luna-Nácar et al., 2016) were used for pathway analysis, BioVenn was used to construct Venn-diagram.

### Flow Cytometry

Cytoflex (Beckman Coulter) flow cytometer was used to quantitate cells. Cells stained with CFW were quantitated by using excitation of 405 nm laser and the fluorescence emission was collected using 450/45 filter. FDA and Alexafluor488-WGA were excited using a 488 nm laser and fluorescence was collected using 525/40 filter. The events/sec passing through the laser was restricted to <500 with a liquid flow rate of 10 μl/s and a total of 10,000 P1 events were captured. After the cells were treated with staining solutions, the flow cytometry analysis was completed within 1 h; the stability of the samples was confirmed by reproducibility of data. At each time point, the appropriate voltage and gates were set by using unstained samples as control. The obtained data were analyzed using CytExpert software (Beckman Coulter).

### Transmission Electron Microscopy

Trophozoites from 24 h culture and enriched cyst-like structures were harvested, washed three times with PBS pH 7.4 and centrifuged at 2,000 *g*, for 5 min. The pelleted cells were re-suspended overnight in 4% glutaraldehyde in 0.1 M sodium cacodylate buffer, pH 7.3 at 4°C. Next day cells were washed thoroughly with cacodylate buffer and post-fixed for 30 min in 1% osmium tetroxide in cacodylate buffer. The fixed cells were dehydrated in progressive concentrations of ethanol (20, 30, 50, 70, 90, 100%) for 10 min each and embedded in LR White resin (medium grade 707047 Sigma Aldrich) and polymerized at 64°C for 40 h in gelatin capsules. Ultrathin sections of 70 nm were cut using an ultramicrotome, placed on copper grid of 300 mesh (SPI 2030C), contrasted with uranyl acetate and viewed using a transmission electron microscope at 43,000 X magnification.

### Clinical Sample Collection

Samples were collected from 22 women of age group 25–45 years, showing symptoms of genitourinary infections attending Obstetrics and Gynecology departments in a private hospital in Bangalore, India. Vaginal discharge was collected from the patient vagina using a sterile swab into culture tubes containing TYI-S33 medium. Microscopy was performed immediately after collecting swab samples by using CFW staining. The swab samples were cultured at 37°C in TYI-S33 medium and checked daily for motility of the parasites under microscope.

### Ethics

Institute Human Ethics Committee (IHEC) reviewed and approved the project (IHEC No: 8b-15032017). Vaginal swabs were collected as per the principles, guidelines and methods approved by IHEC by a trained gynecologist. Patient anonymity was maintained throughout the experiment.

### Statistical Analysis

Results were reported as Mean ± SD. Grouped data was statistically analyzed by two-way ANOVA. For paired comparisons, two-tailed P-test was used. All statistical analysis were performed using GraphPad Prism 5.0.

## Results

### *T. vaginalis* Undergoes Encystation on Prolonged Growth in the Culture Medium

**Figure 1:**
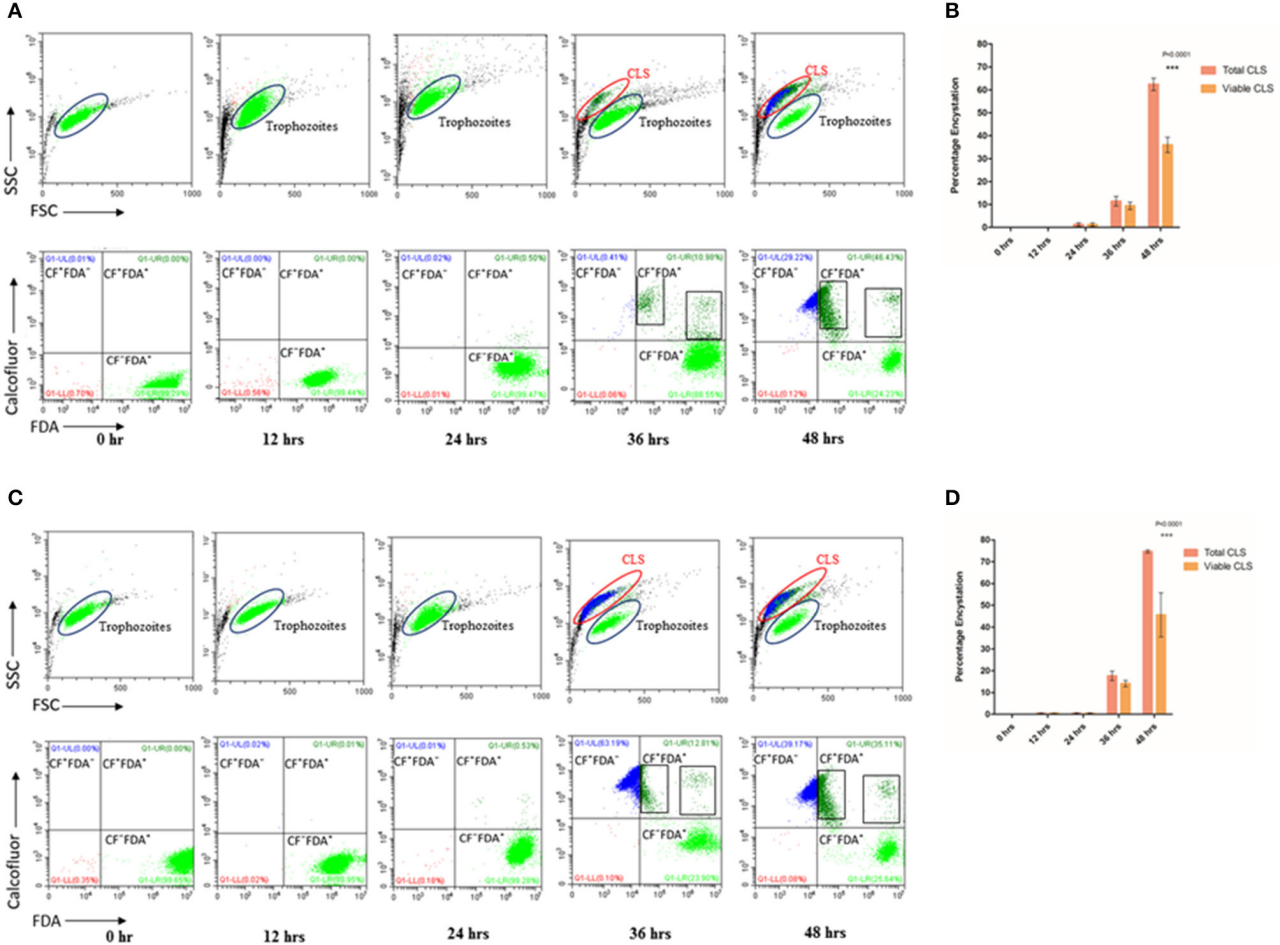
Encystation of trophozoites to cyst-like structures in *Trichomonas vaginalis* by flow cytometric analysis. **(A,C)** *T. vaginalis* trophozoites isolate-1 and *T. vaginalis* clinical isolate-2, respectively were analyzed every 12 h up to 60 h. A new population of Cyst-like structures (CLS; marked in Blue) is observed in FSC vs. SSC graph after 36 h time point which is distinct from the trophozoites (marked in Green). The CFW stained cells are CLS, CFW^+^ FDA^+^ were considered as viable CLS, and CFW^−^ FDA^+^ as trophozoites. **(B,D)** Bar graph showing percentage encystation by flow cytometric quantitation of *T. vaginalis* isolate-1 and *T. vaginalis* Clinical isolate-2 at 12, 24, 36, and 48 h, respectively. Representative data are shown from three independent experiments.

*Trichomonas vaginalis* is believed to occur exclusively in the motile, infective trophozoite form (Schwebke and Burgess, 2004). However, recent reports have pointed out the presence of pseudocysts in the parasite. In our study, we observed that *T. vaginalis* occurs as CLS under conditions of prolonged growth and consequent nutrition depletion. We allowed *T. vaginalis* to grow up to 60 h without changing the growth medium. Every 12 h, cells were stained with Calcofluor White (CFW) and Fluorescein Diacetate (FDA). These were viewed under a fluorescence microscope and flow cytometry was performed for quantitation (Figures 1A,B, Supplementary Figure 1). Till 24 h, all the cells appeared pear-shaped, flagellated, motile, and did not stain with CFW (Supplementary Figure 1A). Beyond 30 h, we observed CLS which stained with CFW (Supplementary Figure 1B). Most of the CLS had internalized flagella, as previously reported (Dias-Lopes et al., 2017), were non-motile and spherical. With increasing time, the percentage of CLS increased in the *in vitro* culture (Supplementary Figure 1C). The experiment was terminated at 60 h. To further confirm presence of chitin exclusively in CLS, we used Alexafluor488 conjugated wheat germ agglutinin (WGA) (Supplementary Figure 6). WGA is a lectin and uniquely binds to N-acetyl glucosamine, which is the building block of chitin. WGA binding assay has been classically used to detect chitin (Ratner et al., 2008; Aguilar-Díaz et al., 2010; Liedke et al., 2017). While enriched CLS showed green fluorescence, trophozoites did not take up the stain. This further strengthened our observation of presence of chitin in *T. vaginalis* CLS and its absence from *T. vaginalis* trophozoites.

To quantitate the conversion of trophozoite to CLS, we considered the CFW stained CLS as total CLS and FDA positive CFW stained cells as viable CLS. *T. vaginalis* has been shown to possess esterases which may release free fluorescein (Aguilar-Díaz et al., 2010) and can be visually seen inside cells (Supplementary Figure 4) CLS was stained blue with CFW indicating presence of chitin in these forms. FSC vs. SSC flow cytometry plot highlights differences based on cell shape, size and granularity in a heterogeneous cell population. As shown in Figure 1A, two distinct cell populations were observed. As compared to trophozoites, CLS showed reduced forward scattering and slightly increased side scattering. Hence, morphological differences between the two forms can be applied to differentiate between *T. vaginalis* trophozoites and CLS forms. Only trophozoite form was present in the logarithmic phase of culture i.e., ~24 h. At 30 h, we observed 11.430 ± 2.080% total CLS (*n* = 3, *P* = 0.0006) and 9.410 ± 1.644% viable CLS as also shown in Figure 1A, using flow cytometry and microscopy as shown in Supplementary Video 1. As shown in Figure 1A, the percentage of total CLS increased to 62.407 ± 2.707 and viable CLS to 36.0 ± 3.336% at 48 h (*n* = 3, *P* = 0.0006). The observation indicates that *T. vaginalis* trophozoites undergo conversion to CLS under conditions of nutritional deprivation. This is in agreement with previous studies reporting formation of cysts under adverse environmental conditions. The fresh clinical isolate also showed a similar process of encystation (Figures 1C,D) in which 17.633 ± 2.178% total CLS and 14.040 ± 1.437% viable CLS were observed at 36 h and 74.657 ± 0.670% total CLS and 45.60 ± 10.101% viable CLS were observed at 48 h.

### *T. vaginalis* CLS Are Resistant to Environmental Stress

**Figure 2:**
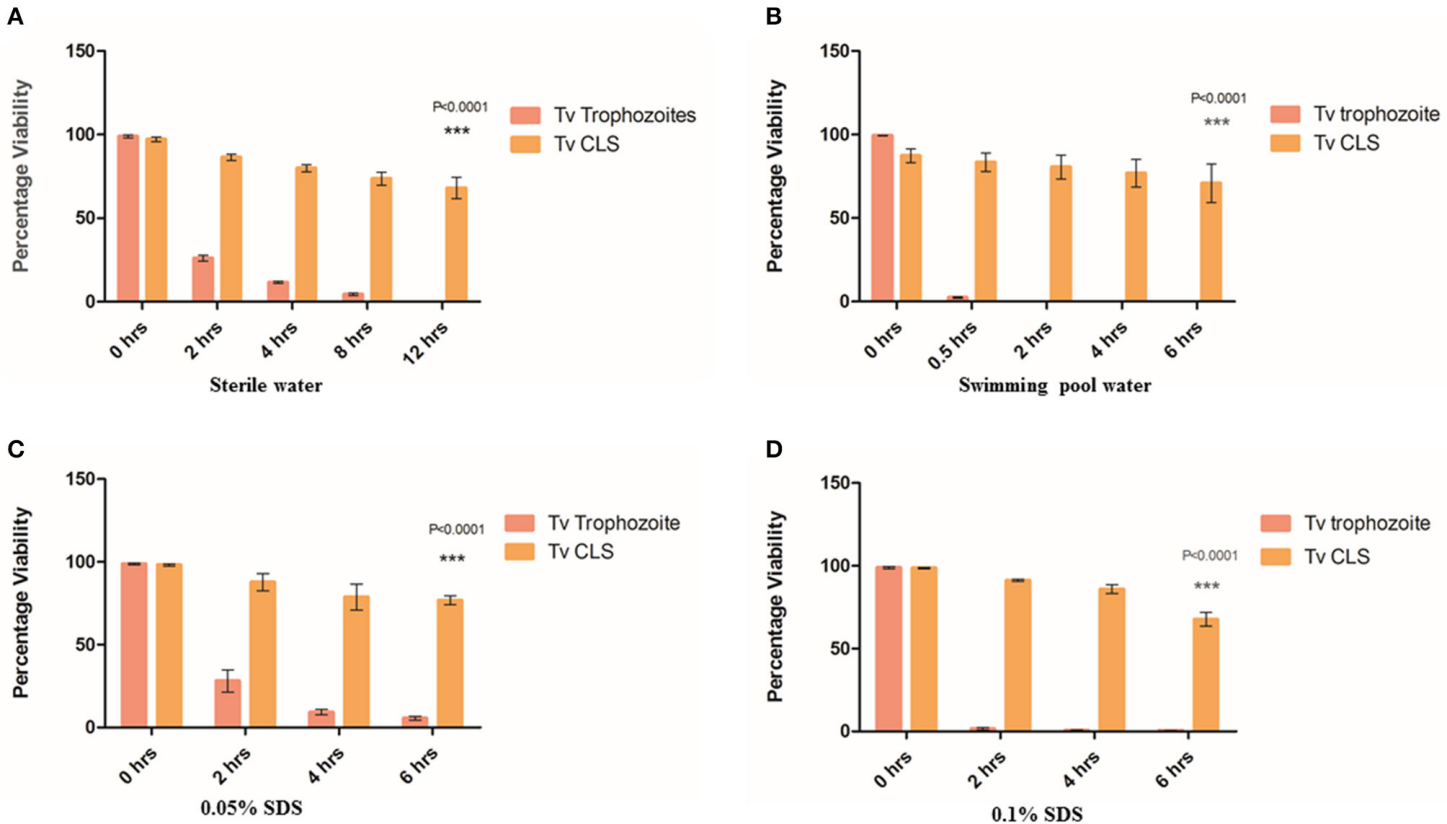
Resistance of cyst-like structures (CLS) to osmotic imbalance and detergent treatments. **(A)** Equal number of pure trophozoites and CLS were incubated in sterile water and their viability was tested at 0, 2, 4, 8, and 12 h. Flow cytometric quantitation was done at each indicative time point for FDA based-viability test. Post 8 h, no trophozoites were observed while >65% CLS remained viable even at 12 h (*P* = < 0.0001; *n* = 3 for each set). **(B)** Equal number of pure trophozoites and enriched CLS were incubated in swimming pool water and viability was tested, as before. Beyond 2 h, trophozoites lysed but >80% CLS were viable till 6 h (*P* < 0.0001; *n* = 3). **(C)** Equal number of pure trophozoites and enriched CLS were incubated in 0.05% SDS and viability was checked at 2, 4, and 6 h. Trophozoites were lysed by detergent but CLS retained >80% viability even after 6 h of treatment (*P* < 0.0001; *n* = 3). **(D)** Similarly, equal number of pure trophozoites and pure CLS were incubated in 0.1% SDS and viability checked at 2, 4, and 6 h. At 6 h, >75% CLS retained their viability (*P* < 0.0001; *n* = 3).

Cysts from other unicellular organisms are known to be resistant to osmotic imbalances (Lauwaet et al., 2007; Aguilar-Díaz et al., 2010). To investigate whether *T. vaginalis* CLS is resistant to osmotic variation in their environment, we first obtained enriched CLS (see Supplementary Figure 2 and section Materials and Methods). 2 × 10^6^ CLS were resuspended in sterile water. As control, equal number of log-phase trophozoites (2 × 10^6^) was resuspended in sterile water. The cells were kept at 37°C and examined for cell count and viability using FDA viability assay at 2, 4, 6, and 8 h. At 2 h, 26 ± 1.868% trophozoites and 86.413 ± 1.898% CLS were viable (*n* = 3). At 8 h, only ~6% trophozoites were viable while >70% of the CLS were viable. Beyond 8 h, no trophozoites were observed on microscopic examination. However, even at 12 h >65% of the CLS remained viable and morphologically healthy (*n* = 3). Thus, CLS of *T. vaginalis*, like cysts from other organisms, are resistant to hypo-osmolarity (Figure 2A). To investigate the duration of viability of CLS in water, we incubated enriched CLS at 25°C in sterile water upto 48 h. CLS was stained using both FDA (provides measure of viability) and Propidium Iodide (PI), a dye used to measure dead cells. Using flow cytometry, we observed 25 and 15% FDA-stained viable CLS at 24 and 48 h, respectively (Supplementary Figure 5). PI was taken up by non-viable CLS (~75% at 24 h and ~85% at 48 h). Thus, at room temperature in conditions of hypo-osmolarity, *T. vaginalis* CLS can remain viable upto 48 h.

Further, we investigated the effect of detergent on viability of CLS as cysts from other organisms are known to be resistant to detergents (Aguilar-Díaz et al., 2010). We used two concentrations of sodium dodecyl sulfate (SDS); 0.05% (Set 1) and 0.1% (Set 2) and investigated the cell numbers and viability at 2, 4, and 6 h. Each set consisted of 2.5 × 10^6^ pure trophozoites and enriched CLS. As shown in Figure 2C, in Set 1, at 2 h, about 20% trophozoites were viable (*n* = 3) while >85% CLS retained viability with no statistically significant change from 0 h (*n* = 3). At 4 h and beyond, about 5% trophozoites were viable but ~80% CLS maintained viability and were capable of excysting back to trophozoites even after 6 h of detergent treatment. In Set 2 (0.1% SDS), a similar trend was observed. 2.713 ± 0.821% trophozoites were observed at 6 h, CLS number and viability (>65% viable at 6 h time point) did not change with any statistical significance indicating resistance of CLS to the detergent-treatment (*n* = 3). At 2, 4, and 6 h, percentage of viable CLS were 91.247 ± 0.956, 85.910 ± 4.596, 67.603 ± 7.200%, respectively (*n* = 3), as shown in Figure 2D. Thus, like cysts of other organisms, *T. vaginalis* cyst-like structures are resistant to detergent treatment.

A previous clinical report had indicated transmission of *T. vaginalis* via swimming pool water (Pereira-Neves and Benchimol, 2008). As swimming pool water is chlorinated and known to be microbiocidal, we wanted to investigate if *T. vaginalis* trophozoites and CLS retained viability when suspended in it. Toward this, swimming pool water was collected, and free chlorine content was found to be 1.2 ppm. 2 × 10^6^ pure trophozoites (Set 1) and pure CLS (Set 2) were incubated with swimming pool water at 37°C and cell count and viability were tested at 0.5, 2, 4, and 6 h (Figure 2B). At 0.5 h, only ~3% trophozoites in Set 1 (2.4 ± 0.529% cells; *n* = 3) remained viable while ~85% CLS in Set 2 (86.417 ± 9.523% cells; *n* = 3) retained viability. At 2 h, no trophozoites in Set 1 retained viability while >80% CLS in Set 2 (80.567 ± 12.393% cells; *n* = 3) remained viable. At 4 and 6 h no trophozoites were observed in Set 1 but ~70% viable CLS in Set 2 retained their viability (76.770 ± 14.319% cells; *n* = 3 at 4 h and 70.803 ± 19.858 cells; *n* = 3 at 6 h). This indicated that CLS can survive in chlorinated water. Thus, indicating environmental resistance of *T. vaginalis* CLS. We hypothesize that this could provide a possible non-sexual mode of transmission of *T. vaginalis* and explain previous findings of transmission of the parasite via toilet seats and swimming pools (Piekarski and Saathoff, 1973).

### *T. vaginalis* Trophozoites Convert to CLS *in vitro* Upon Exposure to Acidic Vaginal pH

Healthy vaginal pH is between 3 and 5, and growth of *T. vaginalis* is known to increase the pH of the vagina (Gjerdingen et al., 2000). We investigated the fate of *in vitro T. vaginalis* trophozoites and CLS at physiological vaginal pH. Equal number (2 × 10^6^ cells) of pure trophozoites and enriched CLS were grown in TYI-S33 medium pH 6.8 (regular TYI-S33 medium), pH 3.0, and pH 5.0 at 37°C. Post 24 h, cells were stained with CFW, scored for viability and counted.

**Figure 3:**
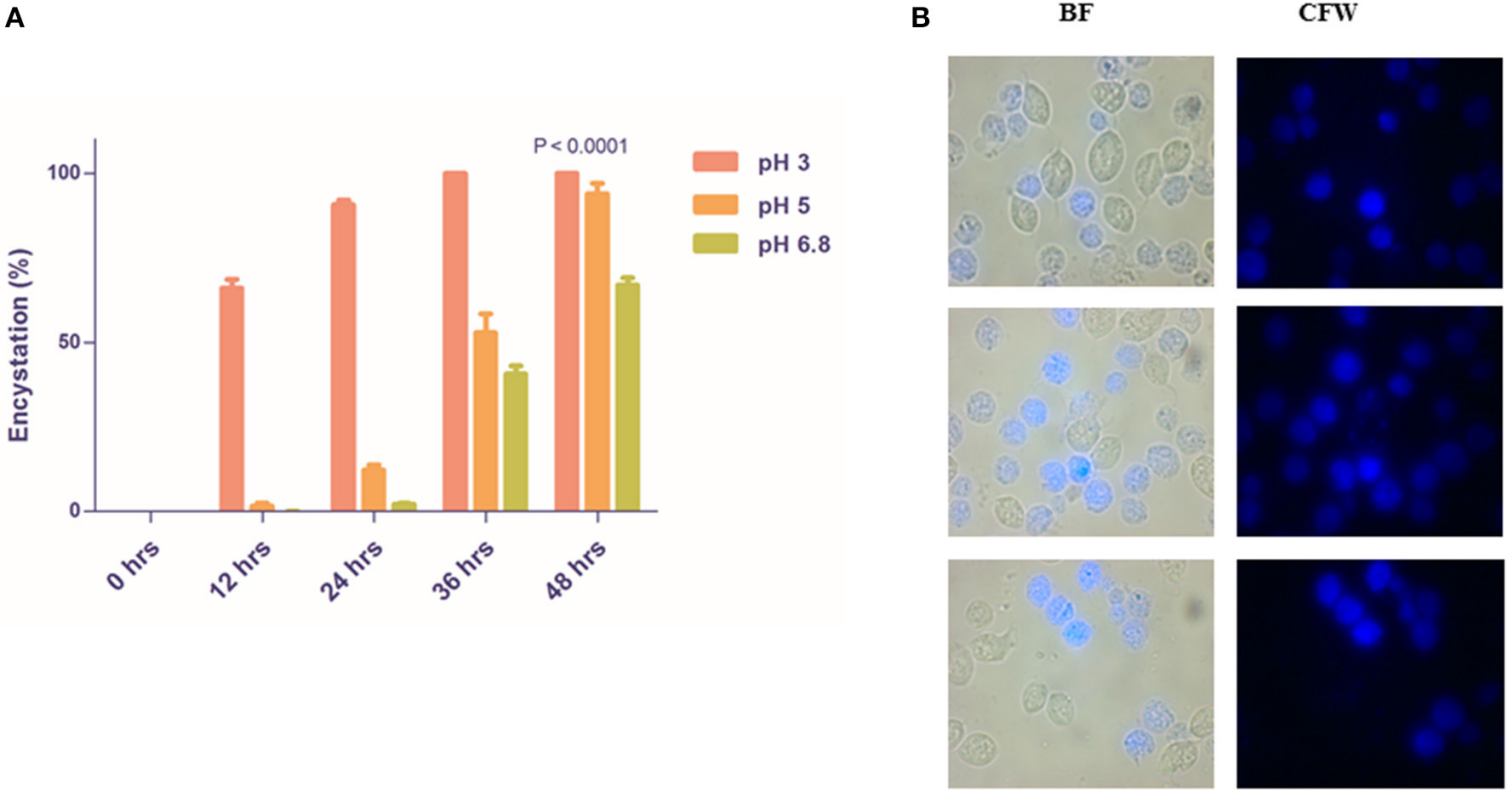
Rapid encystment of *T. vaginalis* trophozoites to CLS at acidic vaginal pH. **(A)** Pure trophozoites were incubated at pH 3, pH 5, and pH 6.8 TYI Medium at 37°C. Percentage encystation of trophozoites to CFW stained CLS at 0, 12, 24, 36, and 48 h was counted. >85% cells encysted to form CLS in pH 3 TYI medium and >50% cells encysted to form CLS in pH 3 TYI medium post 24 h (*P* < 0.0001; *n* = 3 for each data set). **(B)** Post 24 h, the *T. vaginalis* cells grown in TYI medium (pH 5) were observed under microscope. BF, bright field; CFW, calcofluor white stain.

We observed that 66.00 ± 2.646 and 1.6 ± 0.9238% of encystation at 12 h whereas 90.67 ± 1.453 and 12.20 ± 1.562% of encystation at 24 h in medium of pH 3 and pH 5, respectively (Figures 3A,B). More than 40% trophozoites at pH 6.8 maintained normal morphology and motility up to 36 h. CLS in all three pH remained viable and stained with CFW. Our observations demonstrate that trophozoites undergo encystation at physiological pH of the vagina. This is indicative of a possible role of CLS in the parasite's establishment of infection, survival, and pathogenesis under acidic pH conditions.

### *T. vaginalis* CLS Excyst Under Favorable Conditions and Demonstrate Rapid and Enhanced Excystation in Presence of Cervical Cells

**Figure 4:**
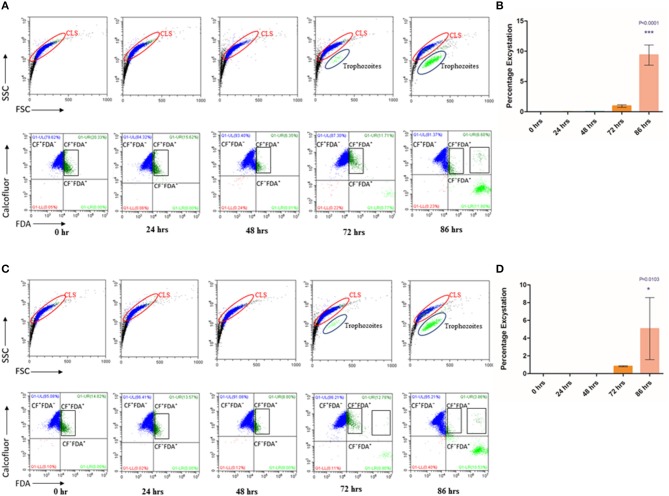
Excystation of CLS to trophozoites in *Trichomonas vaginalis* by flow cytometric analysis. **(A,C)** Enriched CLS were resuspended to fresh TYI medium and analyzed at 24, 48, 72, and 86 h by flow cytometry for viability test for *T. vaginalis* isolate-1 and *T. vaginalis* clinical isolate-2, respectively. At 0 h, all parasites were stained by CFW (marked in Blue). After 48 h, some cells were CFW^−^ FDA^+^ and at 86 h the percentage of CFW^−^ FDA^+^ increased to ~10%, representing the cell population of actively dividing trophozoites (marked in Green). **(B,D)** Bar graph showing percentage excystation of enriched CLS to trophozoites of *T. vaginalis* isolate-1 and *T. vaginalis* clinical isolate-2 quantitated by flow cytometry at 0, 24, 48, 72, and 86 h. Representative data are shown from three independent experiments.

Cysts are known to be formed under unfavorable environmental conditions and revert to trophozoites when conditions become conducive for growth and proliferation (Penfold and Woodcock, 1916; Schaefer et al., 1984). We examined if under favorable conditions, *T. vaginalis* cyst-like structures could excyst to the trophozoite form. Toward this, we obtained enriched CLS (see Methods). Following washes in 1X PBS, we seeded CLS at a concentration of 1 × 10^6^ cells/ml of fresh TYI Medium pH 6.8 and incubated at 37°C. Every 24 h, medium was changed, and cells were examined for cell number, viability and stained with CFW and FDA to calculate percentage excystation and viability, respectively. Up to 48 h, we did not observe excystation, most of the cells were stained with CFW and the FSC vs. SSC plot showed only a single cell population (Figure 4A, Supplementary Figures 3A,B). At 72 h, most cells (>80%) had externalized flagella (Supplementary Figure 3C). A small fraction (0.940 ± 0.203%) of cells did not take up the CFW stain and were FDA positive, were pear-shaped and showed whipping flagellar movement; indicative of their conversion to trophozoites. At 86 h, 9.377 ± 1.665% cells counted had all hallmarks of trophozoites (Figures 4A,B, Supplementary Video 2, Supplementary Figure 3D). The FSC and SSC plots at 72 and 86 h indicate the presence of two distinct populations. The excysted trophozoites were viable and showed normal growth in fresh medium (data not shown). Thus, *T. vaginalis* CLS, like cysts from most other organisms, can excyst under favorable environmental conditions to form viable and actively multiplying trophozoites. The excystation process was observed in the fresh clinical isolate also, with an excystation percentage of 0.833 ± 0.065% at 72 h and 5.067 ± 3.499% at 86 h (Figures 4C,D).

*T. vaginalis* trophozoites are known to disrupt HeLa cells' (cervical cell line) monolayer (Juliano et al., 1991; Gilbert et al., 2000). To investigate the rate of excystation of *T. vaginalis* CLS in presence of host cells, we co-incubated HeLa cells' monolayer with cyst-like structures in ratios of 2:1, 5:1, 10:1, 20:1, and 50:1 (CLS: Hela cells). Similarly, as control, *T. vaginalis* trophozoites were incubated with HeLa cells' monolayer. The cells were maintained in DMEM 10% FBS: TYI Medium in the ratio 3:1 and incubated at 37°C, 5% CO_2_ for 4 h. Pure trophozoites and enriched CLS were also incubated in this medium under the same conditions. Post-incubation, each well was thoroughly washed with 1X PBS and microscopically examined to be devoid of any trophozoite or CLS. The spent medium was pelleted, and cells were stained with CFW and examined under fluorescence microscope. Following this, MTT assay was performed with HeLa cells, as described in section Materials and Methods.

**Figure 5:**
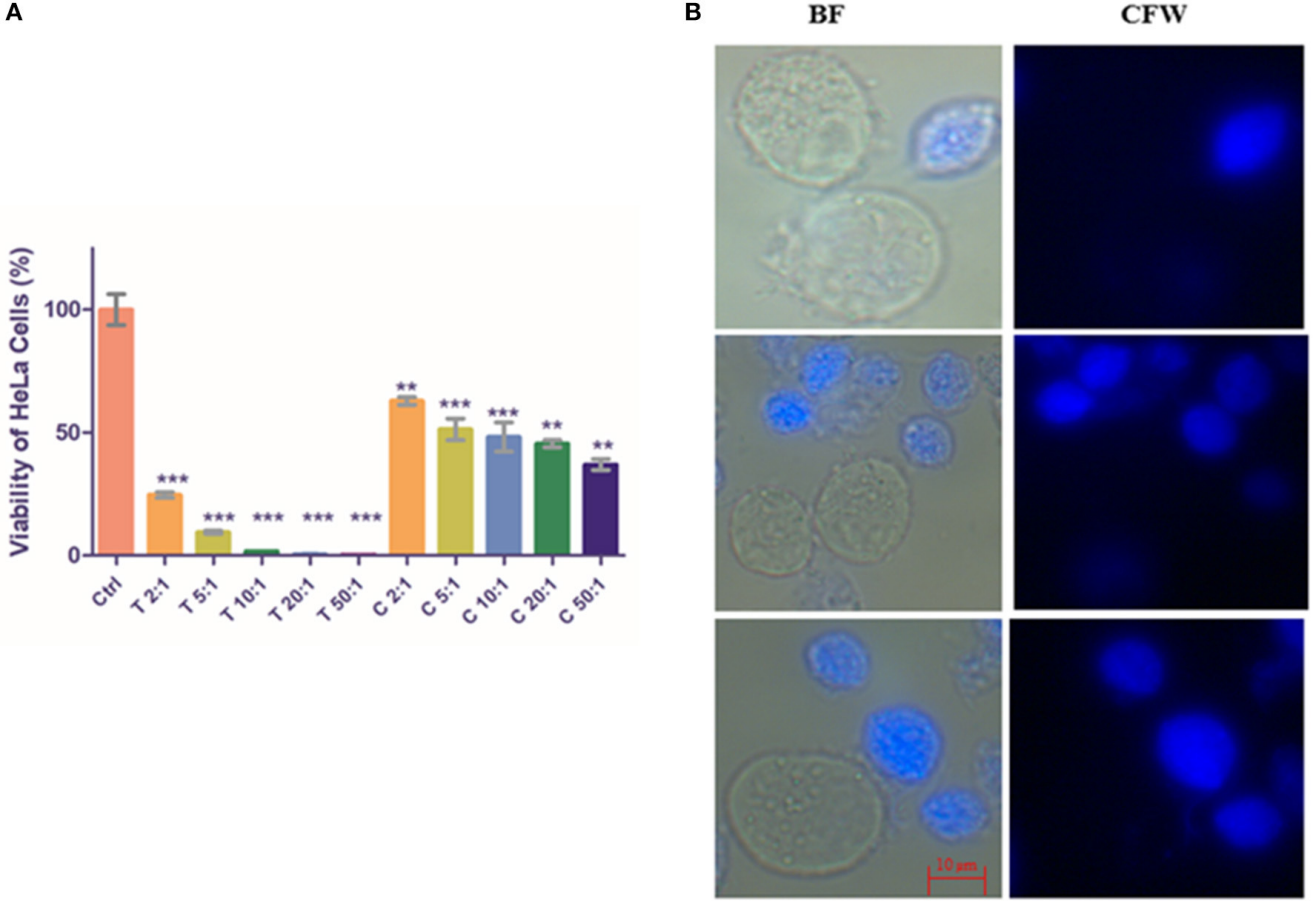
Co-incubation of *T. vaginalis* pure trophozoites and enriched CLS with HeLa cells. **(A)** Pure trophozoites and enriched CLS were co-incubated with a monolayer of HeLa cells in a 24-well plate at ratios of parasite: HeLa cells of 2:1, 5:1, 10:1, 20:1 and 50:1. Post 4 h, parasites were removed and MTT assay was performed for the HeLa cells. As expected, trophozoites lysed the monolayer with viability of HeLa cells <50% even at a 2:1 ratio. Co-incubation with CLS resulted in disruption of the monolayer in a dose-dependent manner with ~40% viability at the highest CLS number of 50:1 (*P* < 0.0001; *n* = 3). **(B)** Trophozoites and CLS co-incubated with HeLa cells were observed under the microscope. In 4 h, a fraction of CLS had excysted to trophozoites, contributing to the virulence of CLS toward HeLa cells. BF, bright field; CFW, calcofluor white stain. Scale bar: 10 μm. **P ≤ 0.01; ***P ≤ 0.001.

As expected, the monolayer of HeLa cells was nearly fully disrupted by *T. vaginalis* trophozoites at ratios of 10:1, 20:1, and 50:1 (Trophozoite: Hela cells; *P* < 0.0001, *n* = 3). As shown in Figure 5A, CLS also exhibited virulence against HeLa cells in a cell number-dependent manner, albeit lesser than pure trophozoites. Cell viability percentage of CLS: HeLa at ratios of 2:1, 5:1, 10:1, 20:1, and 50:1 was observed to be 62.84 ± 1.562 (*P* = 0.0071; *n* = 3), 51.28 ± 4.404 (*P* = 0.0016; *n* = 3), 48.18 ± 5.957 (*P* = 0.0002; *n* = 3), 45.45 ± 1.482 (*P* = 0.0007, *n* = 3), and 36.97 ± 2.23 (*P* = 0.0002, *n* = 3), respectively. Thus, with increase in CLS numbers, an increased number of HeLa cells were disrupted.

Microscopic examination of the parasite in the spent medium post incubation for 4 h revealed that CLS had rapidly excysted to flagellated, motile trophozoites in presence of HeLa cells (Figure 5B). The rate of excystation observed was 15–20%. Our observation suggests that host factors released due to co-incubation of HeLa with CLS play a role in enhancing rate and time of excystation (the effect of DMEM 10% FBS: TYI 3:1 was ruled out as CLS incubated in this medium did not show any morphological changes).

### CLS Coexist With Trophozoites in Clinical Vaginal Swabs of Symptomatic Human Patients

**Figure 6:**
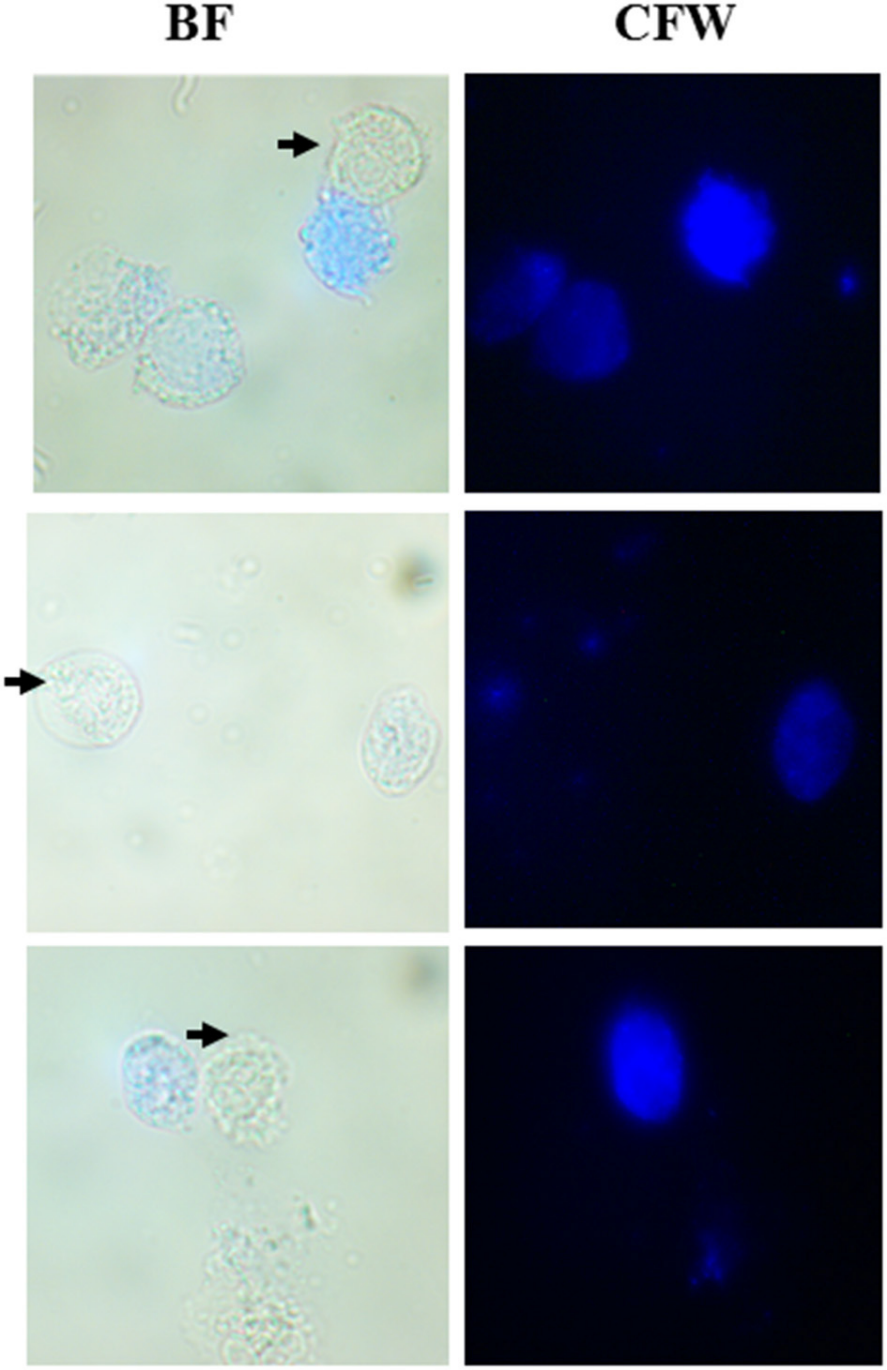
Co-existence of *T. vaginalis* trophozoites and cyst-like Structures in human clinical samples. Human *T. vaginalis* samples tested positive by microscopy showed both the trophozoite (marked with black arrows) and CLS (spherical structures stained with CFW). BF, bright field; CFW, calcofluor white.

As described under section Materials and Methods, we collected vaginal swabs from 22 female patients who presented with symptoms of urogenital infection. When observed under the microscope, out of 22 samples, 3 tested positive for *T. vaginalis*. In all three cases, we observed the co-existence of active and motile trophozoites along with Cyst-like structures which stained blue with CFW (Figure 6). We maintained these parasites in TYI-S33 medium at 37°C. These parasites underwent encystation and the percentage encystation was similar to previously passaged lab culture. Thus, in the clinical scenario, cyst-like structures are present along with trophozoites and may play an important role in pathogenicity and transmission of the disease.

### Proteomic Differences Between *T. vaginalis* Trophozoites and CLS

**Figure 7:**
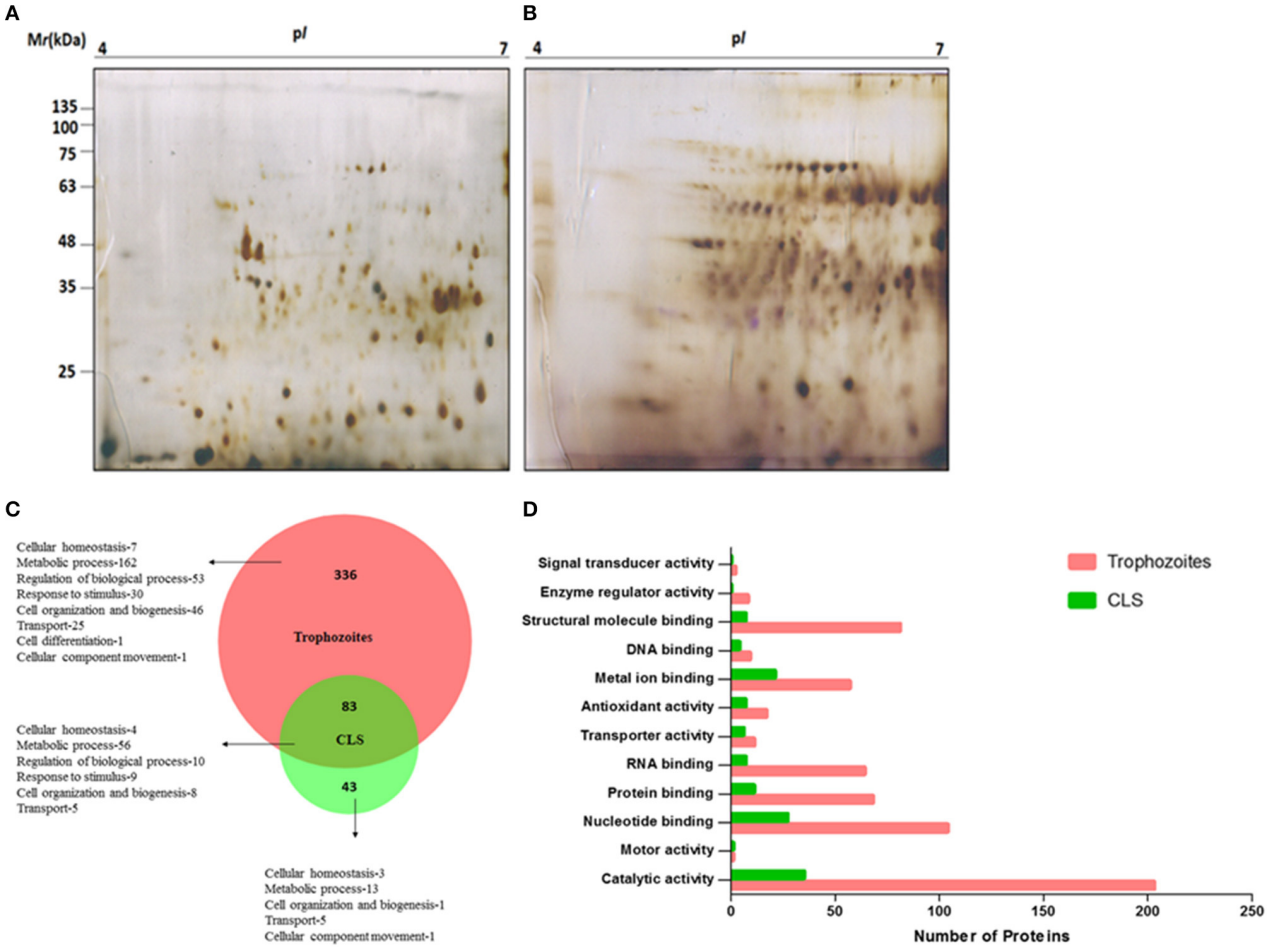
Proteome analysis of *T. vaginalis* trophozoites and CLS. Two-dimensional gel electrophoresis profile of *T. vaginalis* **(A)** trophozoite and **(B)** CLS. The proteins were separated in the first dimension using isoelectric focusing at pH 4–7 and in second dimension on a 12% polyacrylamide gel followed by silver staining **(C)** biological processes of Trophozoites and Cyst-like structures (CLS) proteins represented in the form of Venn diagram. Intersection show shared functions among both the forms **(D)** functional classification of Trophozoites and Cyst-like structures in form of bar graph.

To analyze protein expression profiles of *T. vaginalis* trophozoites and cyst-like structures, soluble proteins extracted from pure trophozoites and enriched CLS were separated by using 2D-GE using IPG strip of pH range 4–7 (as detailed in section Materials and Methods). Majority of the protein spots were present in molecular weight (MW) between 11 and 75 kDa and in pH range 5–7 (Figures 7A,B). Approximately, 180 protein spots were detected in each of the forms. The starkly different protein profiles of the two forms is indicative of differences between the two forms of the parasite.

As described under section Materials and Methods, we performed in solution trypsin digestion of pure trophozoites and enriched CLS proteins followed by LC-MS/MS. Using Proteome Discoverer^TM^, we identified a total of 473 proteins with high confidence and FDR of 0.01, maximum 2 missed cleavage sites and carbidomethylation as the fixed modification (Supplementary File 2). Forty-three proteins were unique to CLS and 336 proteins were unique to trophozoites and 83 were common to both from 3 biological replicates as shown in Figure 7C (Supplementary Files 3, 4). A functional pathway analysis of these unique proteins using Proteome Discoverer revealed that while metabolically active trophozoites expressed proteins for glycolysis, gluconeogenesis, pentose phosphate pathway, amino acid synthesis, one carbon metabolism, nucleotide synthesis and antioxidant defense, CLS expressed proteins related to metal binding, nucleotide binding, structural molecule binding, and catalytic activity (Supplementary Files 3, 4). Reduced number of proteins in CLS reiterate the notion that CLS has lowered metabolism as compared to trophozoites. Our data agrees with the previously published recent study of comparison of proteomes of the two forms (Dias-Lopes et al., 2018) which demonstrates the differences in the metabolic state of pseudocysts formed by iron-depletion in the culture medium. It highlighted the metabolic shift of pseudocysts to a lower glycolytic phenotype as compared to trophozoites and significant alterations in proteins of adhesion and cytoskeleton reorganization. Our analysis confirms these findings and differences in proteomes of the two forms can be further used to understand mechanisms underpinning regulation of CLS formation in *T. vaginalis*.

Using BioVenn, we constructed a Venn diagram representing the different biological process of trophozoite and CLS (Figure 7C). Most proteins identified were enzymes in both the groups. The proteins which did not have GO annotated terms associated in PANTHER were identified by using Argot2 (Supplementary File 5). However, CLS showed reduced expression of antioxidant proteins and number of signal transducer and transporter proteins (Figure 7D). These differences reinstate our previous observations and indicate that *T. vaginalis* has two distinct forms which differ not only in their morphological features, but also exhibit differences at the proteomic level.

### Ultrastructure of *T. vaginalis* Trophozoite and CLS

A thick cyst wall is a hallmark of cysts. To visualize cyst wall, transmission electron microscopy (TEM) is the method of choice. There are several ultrastructural studies of trophozoite form of *Trichomonas* sp., however, very few studies have looked at *T. vaginalis* pseudocysts. A study compared pseudocysts from *T. foetus* and *T. vaginalis* and provided Scanning Electron Microscopic (SEM) as well as TEM images for the same (Pereira-Neves et al., 2003). It was shown that pseudocysts had internalized flagella and a thick electron-dense deposition along the cell membrane which was completely absent in the trophozoite form (Pereira-Neves et al., 2003). Another study in 2012 (Afzan and Suresh, 2012) performed TEM with day 3 culture of *T. vaginalis* pseudocysts collected from patients with and without cervical neoplasia. They observed that pseudocysts from patients with cervical neoplasia had thicker cyst walls. Another study by Dias-Lopes et al. (2017) used iron depletion to form pseudocysts and performed TEM with *T. vaginalis* trophozoites and newly formed (within minutes) pseudocysts. However, the membrane thickness observed by them was comparable to that of trophozoites. Thus, there seems to be a disparity in the thickness of cyst wall obtained among different studies. Like related parasites (Frisardi et al., 2000) this may be attributed to the stage of cyst-formation; while earlier cysts may show thinner cyst walls, mature cysts may have thicker cyst walls.

**Figure 8:**
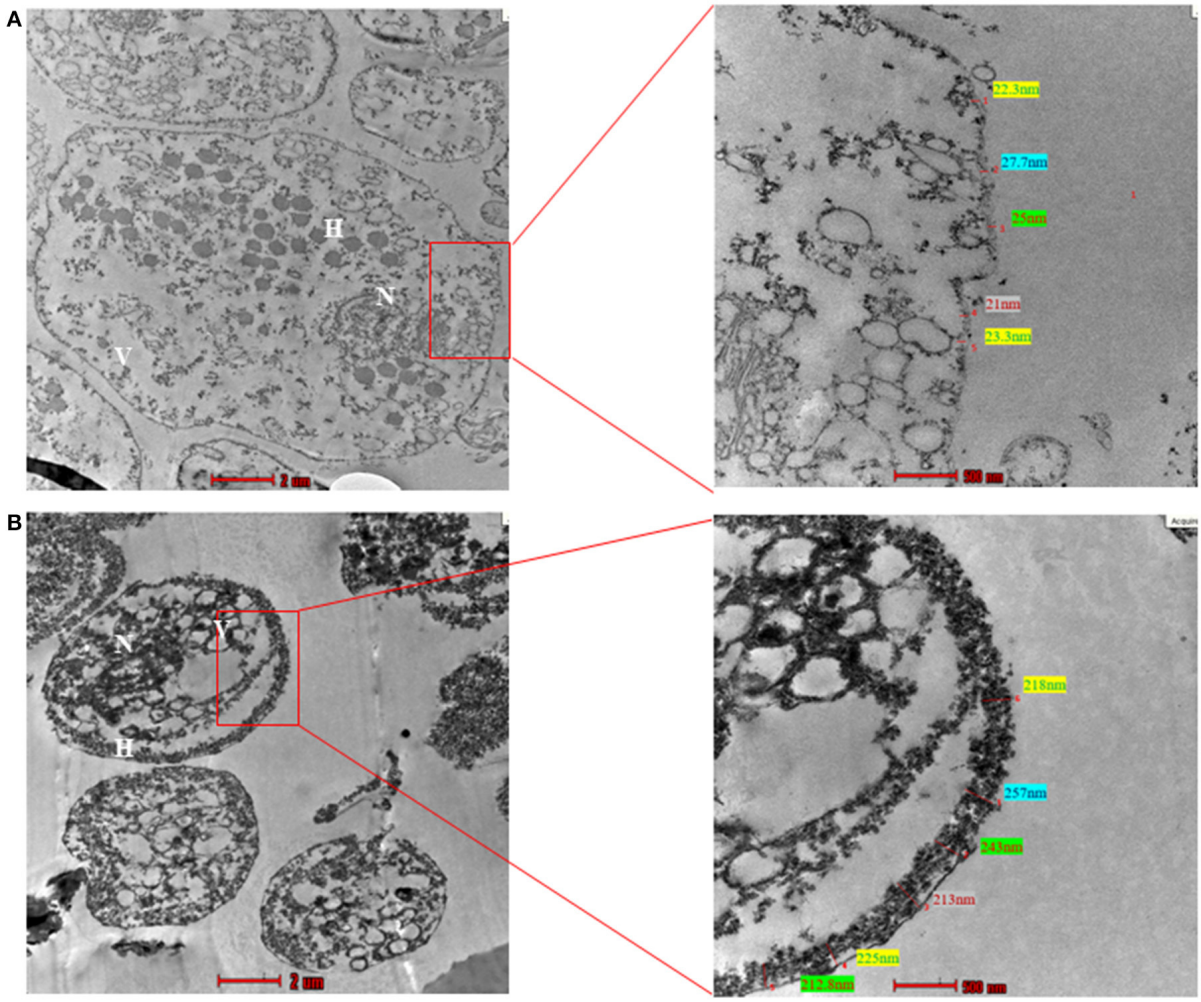
Transmission Electron Microscopy (TEM) of trophozoite and Cyst-like structures of *T. vaginalis* (magnification X 43,000). **(A)** The image shows the transmission electron micrograph of trophozoites. The cell membrane presented thickness ranging between 9.7 and 27.2 nm. **(B)** The transmission electron micrograph of CLS of *T. vaginalis* reveals deposition of presumably chitin along outer membrane ranging from 230 to 257 nm. N, nucleus; F, flagella; H, hydrogenosomes.

To examine the ultrastructure of CLS obtained in our experiments, we resorted to TEM. We used pure trophozoites and enriched CLS to capture TEM images. The CLS used in this imaging was obtained by growing *T. vaginalis* culture for 48 h, followed by enrichment in sterile water for 12 h. As previously shown by light microscopy, transmission electron micrographs revealed CLS to be spherical and smaller in size as compared to the trophozoite form (Figure 8). The nucleus of trophozoite showed less condensed chromatin and distinct hydrogenosomes and vacuoles. A thin outer membrane ranging from 9.7 to 27.2 nm, was observed. On the other hand, ultrastructure of CLS revealed a condensed mass of chromatin and a thick deposition of presumably chitin along its outer membrane of thickness ranging from 230 to 257 nm, which is more than observed in pseudocysts from cervical neoplasia patient (ranged upto 28.01 nm) (Afzan and Suresh, 2012). The external flagella were present only in trophozoite forms of *T. vaginalis*. There were distinguishing characteristics present in nucleus, vacuole size, cell shape, and cell size in both the forms. Thus, the average thickness of outer membrane observed in trophozoite was 17.7 ± 2 nm and CLS was 242 ± 2.5 nm (10 cells per form Supplementary Figure 7). There is deposition of presumably chitin along the outer membrane in cyst-like structures which was absent in trophozoites. Though the cyst wall of *T. vaginalis* CLS is not as thick as cysts from *G. lamblia* (300 nm) (Sun et al., 2003) and *Toxoplasma gondii* cyst wall membrane (upto 850 nm) (Zhang et al., 2010), it appears that it substantially protects the parasite from environmental stressful conditions and maintains viability in these extreme conditions.

## Discussion

*Trichomonas vaginalis* is a clinically important but neglected parasite. Its cosmopolitan distribution across all racial groups and socio-economic strata around the world makes it the most common non-viral STD (De Waaij et al., 2017; Gatti et al., 2017). Despite its clinical and socio-economic relevance, it is a neglected disease due to its higher prevalence in women, social taboo associated with STDs and the paucity of research in this field.

Most studies in *T. vaginalis* have emphasized on the trophozoite form. Pseudocysts have been described in Trichomonads as morphologically transformed non-motile forms without a true cyst wall (Clark, 2003). Some past reports have suggested them to be degenerative forms (Petrin et al., 1998). True cyst wall had been reported in close relatives of *T. vaginalis*: intestine-dwelling trichomonads: *Trichomitus batachorum, Trichomitus sanguisugae*, and free-living *DiTrichomonas honigbergii* (Pereira-Neves et al., 2003). Recent studies on related *T. foetus* have emphasized the importance of this form in disease pathogenesis and non-sexual transmission. Further, the presence of *T. vaginalis* in cervical neoplasia patients and in vagina of rats clearly indicate the importance of cyst like forms in the parasite's life cycle (Hussein and Atwa, 2008). Interestingly, non-sexual modes of parasite transmission have also been reported but not thoroughly investigated (Piekarski and Saathoff, 1973; Pereira-Neves and Benchimol, 2008) in *T. vaginalis*.

In light of these facts, we investigated the presence of *T. vaginalis* cyst-like structures (CLS). We successfully established encystation of trophozoites to CLS in the stationary phase of the *in vitro* axenic culture and excystation of CLS back to trophozoites in fresh medium. Further, we enriched the CLS experimentally by osmotic lysis of remaining trophozoites. This could provide excellent opportunities to study each of the distinct forms and identify molecular differences between them. The CLS of *T. vaginalis* (a) has a chitin-containing outer covering (as shown by staining of CFW, WGA) (b) is resistant to hypo-osmolarity (c) is resistant to detergent treatment (d) can survive when faced with stresses like nutritional deficiency on prolonged growth, chlorinated water treatment and low pH (e) under favorable environment, can revert to trophozoites (f) metabolically quiescent compared to trophozoites as shown by a previous study and our proteomics analysis (g) showed deposition of electron-dense structure along the outer membrane with thickness comparable to cyst wall of related parasites (as seen in TEM images); and (h) is present in clinical samples of female vaginal swabs; and is thus, clinically relevant. Interestingly, the parasite encodes and expresses all the genes belonging to the chitin biosynthesis pathway (data not shown) which further confirms our observation of presence of chitin in *T. vaginalis* CLS. We thus propose nomenclature of these forms as CLS, rather than pseudocysts since these forms exhibit features similar to other parasitic protozoan cysts like *E. histolytica* and *G. lamblia* (Arroyo-Begovich et al., 1980; Ward et al., 1985).

Further, we have described the encystation of trophozoites in acidic pH conditions which is similar to the environment faced by the parasite in the vagina. Our study indicated that low vaginal pH could act as a cue for the parasite to convert from trophozoite to CLS. It is well-known that infection of *T. vaginalis* leads to increase of pH in the vagina due to changes in the vaginal microbiome from acid-producing *Lactobacillus* sp. to anaerobic bacteria (Ward et al., 1985). Thus, perhaps initially at pH 3-5, CLS play an important role in establishing infection and once the pH of the vagina is increased, CLS excyst to trophozoites which are the more virulent form of the parasite. Further, we have shown that interaction of CLS with HeLa cells, a cervical cell line, leads to rapid excystation to trophozoites and consequent lysis of the HeLa cells. Further, our study demonstrated that CLS can survive in chlorinated water; and thus, may play an important role in non-sexual mode of transmission of *T. vaginalis*, as also implicated by a previous study (Pereira-Neves and Benchimol, 2008). Additionally, our mass spectrometry data showed major differences between the proteomes of CLS and trophozoites and highlights the different metabolic states of the two forms. The presence of a thick outer membrane as shown by transmission electron microscopy highlights the capability of cyst-like structures to survive under adverse conditions. The presence of both Cyst-like structures and trophozoites in human patient samples demonstrates the importance of cyst-like forms in the clinical scenario. In conclusion, CLS may play an essential role in establishment of initial infection under acidic pH conditions and could aid in non-sexual mode of transmission of the parasite. The possibility of non-sexual mode of transmission can have a far-reaching impact on the epidemiology of the disease and warrants an additional level of caution to be exercised by clinicians and patients.

## Data Availability Statement

The raw data supporting the conclusions of this manuscript will be made available by the authors, without undue reservation, to any qualified researcher.

## Ethics Statement

The studies involving human participants were reviewed and approved by Institute Human Ethics Committee. The patients/participants provided their written informed consent to participate in this study.

## Author Contributions

DB and UT conceived and designed the study. DB and PY performed the experiments. DG and CN performed mass spectrometry. HD procured clinical human samples. DB, PY, and UT analyzed the results and wrote the manuscript. All authors reviewed the manuscript.

### Conflict of Interest

HD was employed by Indira Clinic. The remaining authors declare that the research was conducted in the absence of any commercial or financial relationships that could be construed as a potential conflict of interest.

## Supplementary Material

Supplementary Methods Liquid Chromatography and Mass spectrometry parameters for proteome analysis of *T. vaginalis* trophozoite and CLS.

Supplementary File 1 Supplementary Figures 1–7.

Supplementary File 2 List of all proteins identified by mass spectrometry in trophozoite and CLS of *T. vaginalis*.

Supplementary File 3 List of proteins unique to *T. vaginalis* trophozoites and their related pathways.

Supplementary File 4 List of proteins unique to *T. vaginalis* cyst-like structures (CLS) and their related pathways.

Supplementary File 5 List of proteins of *T. vaginalis* trophozoite and CLS by ARGOT2.

Supplementary Video 1 Video of *T. vaginalis* trophozoites and CLS (shown by blue CFW stain) at 30 h of encystation.

Supplementary Video 2 Video of *T. vaginalis* trophozoites and CLS (shown by blue CFW stain) at 86 h of excystation.

## References

[B1] Afzan M. Y. Suresh K. (2012). Pseudocyst forms of *Trichomonas vaginalis* from cervical neoplasia. Parasitol. Res. 111, 371–381. 10.1007/s00436-012-2848-3 22398830

[B2] Aguilar-Díaz H. Diaz-Gallardo M. Laclette J. P. Carrero J. C. (2010). *In vitro* induction of *Entamoeba histolytica* cyst-like structures from trophozoites. PLoS Negl. Trop. Dis. 4:e607. 10.1371/journal.pntd.0000607 20169067 PMC2821915

[B3] Arroyo-Begovich A. Cárabez-Trejo A. Ruíz-Herrera J. (1980). Identification of the structural component in the cyst wall of *Entamoeba invadens*. J. Parasitol. 66, 735–741. 10.2307/3280662 7463242

[B4] Chatterjee A. Ghosh S. K. Jang K. Bullitt E. Moore L. Robbins P. W.. (2009). Evidence for a “wattle and daub” model of the cyst wall of entamoeba. PLoS Pathog. 5:e1000498. 10.1371/journal.ppat.1000498 19578434 PMC2698119

[B5] Clark H. M. (2003). Neuromuscular treatments for speech and swallowing: a tutorial. Am. J. Speech Lang. Pathol. 12, 400–415. 10.1044/1058-0360(2003/086) 14658992

[B6] De Waaij D. J. Dubbink J. H. Ouburg S. Peters R. P. H. Morré S. A. (2017). Prevalence of *Trichomonas vaginalis* infection and protozoan load in South African women: a cross-sectional study. BMJ Open 7:e016959. 10.1136/bmjopen-2017-016959 28993385 PMC5640031

[B7] Demes P. Gombosova A. Valent M. Fabusova H. Janoska A. (1988). Fewer *Trichomonas vaginalis* organisms in vaginas of infected women during menstruation. Genitourin. Med. 64, 22–24. 10.1136/sti.64.1.22 3257936 PMC1194141

[B8] Dias-Lopes G. Saboia-Vahia L. Margotti E. T. Fernandes N. S. Castro C. L. F. Oliveira F. O.. (2017). Morphologic study of the effect of iron on pseudocyst formation in *Trichomonas vaginalis* and its interaction with human epithelial cells. Mem. Inst. Oswaldo Cruz 112, 664–673. 10.1590/0074-02760170032 28953994 PMC5607515

[B9] Dias-Lopes G. Wiśniewski J. R. De Souza N. P. Vidal V. E. Padrón G. Britto C.. (2018). In-depth quantitative proteomic analysis of trophozoites and pseudocysts of *Trichomonas vaginalis*. J. Proteome Res. 17, 3704–3718. 10.1021/acs.jproteome.8b00343 30239205

[B10] Dunne R. L. Dunn L. A. Upcroft P. O'donoghue P. J. Upcroft J. A. (2003). Drug resistance in the sexually transmitted protozoan *Trichomonas vaginalis*. Cell Res. 13, 239–249. 10.1038/sj.cr.7290169 12974614

[B11] Frisardi M. Ghosh S. K. Field J. Van Dellen K. Rogers R. Robbins P.. (2000). The most abundant glycoprotein of amebic cyst walls (Jacob) is a lectin with five Cys-rich, chitin-binding domains. Infect. Immun. 68, 4217–4224. 10.1128/iai.68.7.4217-4224.2000 10858239 PMC101730

[B12] Gatti F. A. Ceolan E. Greco F. S. Santos P. C. Klafke G. B. De Oliveira G. R.. (2017). The prevalence of trichomoniasis and associated factors among women treated at a university hospital in southern Brazil. PLoS ONE 12:e0173604. 10.1371/journal.pone.0173604 28346531 PMC5367685

[B13] Gilbert R. O. Elia G. Beach D. H. Klaessig S. Singh B. N. (2000). Cytopathogenic effect of *Trichomonas vaginalis* on human vaginal epithelial cells cultured *in vitro*. Infect. Immun. 68, 4200–4206. 10.1128/iai.68.7.4200-4206.2000 10858237 PMC101726

[B14] Gjerdingen D. Fontaine P. Bixby M. Santilli J. Welsh J. (2000). The impact of regular vaginal pH screening on the diagnosis of bacterial vaginosis in pregnancy. J. Fam. Pract. 49, 39–43. 10678339

[B15] Hirt R. P. (2013). *Trichomonas vaginalis* virulence factors: an integrative overview. Sex. Transm. Infect. 89, 439–443. 10.1136/sextrans-2013-051105 23694938 PMC3749517

[B16] Huang Da W. Sherman B. T. Lempicki R. A. (2009). Systematic and integrative analysis of large gene lists using DAVID bioinformatics resources. Nat. Protoc. 4, 44–57. 10.1038/nprot.2008.211 19131956

[B17] Hussein E. M. Atwa M. M. (2008). Infectivity of *Trichomonas vaginalis* pseudocysts inoculated intra-vaginally in mice. J. Egypt. Soc. Parasitol. 38, 749–762. 19209760

[B18] Johnston V. J. Mabey D. C. (2008). Global epidemiology and control of *Trichomonas vaginalis*. Curr. Opin. Infect. Dis. 21, 56–64. 10.1097/QCO.0b013e3282f3d999 18192787

[B19] Juliano C. Cappuccinelli P. Mattana A. (1991). *In vitro* phagocytic interaction between *Trichomonas vaginalis* isolates and bacteria. Eur. J. Clin. Microbiol. Infect. Dis. 10, 497–502. 10.1007/bf01963936 1915384

[B20] Kawamoto F. Mizuno S. Fujioka H. Kumada N. Sugiyama E. Takeuchi T.. (1987). Simple and rapid staining for detection of Entamoeba cysts and other protozoans with fluorochromes. Jpn. J. Med. Sci. Biol. 40, 35–46. 10.7883/yoken1952.40.35 3626134

[B21] Kneipp L. F. Andrade A. F. De Souza W. Angluster J. Alviano C. S. Travassos L. R. (1998). *Trichomonas vaginalis* and *Tritrichomonas foetus*: expression of chitin at the cell surface. Exp. Parasitol. 89, 195–204. 10.1006/expr.1998.4290 9635443

[B22] Lauwaet T. Davids B. J. Reiner D. S. Gillin F. D. (2007). Encystation of *Giardia lamblia*: a model for other parasites. Curr. Opin. Microbiol. 10, 554–559. 10.1016/j.mib.2007.09.011 17981075 PMC2709507

[B23] Liedke S. C. Miranda D. Z. Gomes K. X. Gonçalves J. L. S. Frases S. Nosanchuk J. D.. (2017). Characterization of the antifungal functions of a WGA-Fc (IgG2a) fusion protein binding to cell wall chitin oligomers. Sci. Rep. 7:12187. 10.1038/s41598-017-12540-y 28939893 PMC5610272

[B24] Luna-Nácar M. Navarrete-Perea J. Moguel B. Bobes R. J. Laclette J. P. Carrero J. C. (2016). Proteomic study of *Entamoeba histolytica* trophozoites, cysts, and cyst-like structures. PLoS ONE 11:e0156018. 10.1371/journal.pone.0156018 27228164 PMC4882050

[B25] Mi H. Muruganujan A. Casagrande J. T. Thomas P. D. (2013). Large-scale gene function analysis with the PANTHER classification system. Nat. Protoc. 8, 1551–1566. 10.1038/nprot.2013.092 23868073 PMC6519453

[B26] Newman L. Rowley J. Vander Hoorn S. Wijesooriya N. S. Unemo M. Low N.. (2015). Global estimates of the prevalence and incidence of four curable sexually transmitted infections in 2012 based on systematic review and global reporting. PLoS ONE 10:e0143304. 10.1371/journal.pone.0143304 26646541 PMC4672879

[B27] Penfold W. J. Woodcock H. M. (1916). The excystation of *Entamoeba histolytica* (Tetragena) as an indication of the vitality of the cysts. Br. Med. J. 1, 714–715. 10.1136/bmj.1.2890.714 20768140 PMC2347513

[B28] Pereira-Neves A. Benchimol M. (2008). *Trichomonas vaginalis: in vitro* survival in swimming pool water samples. Exp. Parasitol. 118, 438–441. 10.1016/j.exppara.2007.09.005 17949719

[B29] Pereira-Neves A. Ribeiro K. C. Benchimol M. (2003). Pseudocysts in trichomonads–new insights. Protist 154, 313–329. 10.1078/143446103322454095 14658492

[B30] Petrin D. Delgaty K. Bhatt R. Garber G. (1998). Clinical and microbiological aspects of *Trichomonas vaginalis*. Clin. Microbiol. Rev. 11, 300–317. 10.1128/CMR.11.2.300 9564565 PMC106834

[B31] Piekarski G. Saathoff M. (1973). [*Trichomonas vaginalis* infections due to use of public swimming pools]. Immun. Infekt. 1, 22–25. 4549630

[B32] Ratner D. M. Cui J. Steffen M. Moore L. L. Robbins P. W. Samuelson J. (2008). Changes in the N-glycome, glycoproteins with Asn-linked glycans, of *Giardia lamblia* with differentiation from trophozoites to cysts. Eukaryot. Cell 7, 1930–1940. 10.1128/EC.00268-08 18820077 PMC2583543

[B33] Ribeiro K. C. Arnholdt A. C. Benchimol M. (2002). Tritrichomonas foetus: induced division synchrony by hydroxyurea. Parasitol. Res. 88, 627–631. 10.1007/s00436-002-0628-1 12107454

[B34] Schaefer F. W. III. Rice E. W. Hoff J. C. (1984). Factors promoting *in vitro* excystation of Giardia muris cysts. Trans. R. Soc. Trop. Med. Hyg. 78, 795–800. 10.1016/0035-9203(84)90024-5 6533854

[B35] Schwebke J. R. Burgess D. (2004). Trichomoniasis. Clin Microbiol. Rev. 17, 794–803. 10.1128/CMR.17.4.794-803.2004 15489349 PMC523559

[B36] Singh M. Beri D. Nageshan R. K. Chavaan L. Gadara D. Poojary M.. (2018). A secreted Heat shock protein 90 of *Trichomonas vaginalis*. PLoS Negl. Trop. Dis. 12:e0006493. 10.1371/journal.pntd.0006493 29768419 PMC5973626

[B37] Sun C. H. McCaffery J. M. Reiner D. S. Gillin F. D. (2003). Mining the *Giardia lamblia* genome for new cyst wall proteins. J. Biol. Chem. 278, 21701–21708. 10.1074/jbc.M302023200 12686559

[B38] Van Meerloo J. Kaspers G. J. Cloos J. (2011). Cell sensitivity assays: the MTT assay. Methods Mol. Biol. 731, 237–245. 10.1007/978-1-61779-080-5_20 21516412

[B39] Walther H. (1973). [Trichomonas infection in clinical picture, therapy and significance to fertility and sterility]. Z. Hautkr. 48, 553–562. 4784128

[B40] Ward H. D. Alroy J. Lev B. I. Keusch G. T. Pereira M. E. (1985). Identification of chitin as a structural component of Giardia cysts. Infect. Immun. 49, 629–634. 4030095 10.1128/iai.49.3.629-634.1985 PMC261227

[B41] Warton A. Honigberg B. M. (1979). Structure of trichomonads as revealed by scanning electron microscopy. J. Protozool. 26, 56–62. 10.1111/j.1550-7408.1979.tb02732.x 314517

[B42] Zarei Z. Mohebali M. Khanaliha K. Kia E. B. Motevalli Haghi A. Heidari Z.. (2018). Detection of pseudocyst forms of *Trichomonas muris* in rodents from Iran. Iran. J. Public Health 47, 729–734. 29922616 PMC6005970

[B43] Zhang Y. W. Halonen S. K. Ma Y. F. Tanowtiz H. B. Weiss L. M. (2010). A purification method for enrichment of the *Toxoplasma gondii* cyst wall. J. Neuroparasitol. 1:N101001. 10.4303/jnp/N101001 21687827 PMC3115730

