# Supplementary material for: Demonstration and Characterization of Cyst-Like Structures in the Life Cycle of Trichomonas vaginalis
Source: Front Cell Infect Microbiol. 2020 Jan 14;9:430. doi: 10.3389/fcimb.2019.00430 (PMC6972724; doi:10.3389/fcimb.2019.00430)
Supplement: Supplementary file 1 [file Data_Sheet_1.PDF]

Supplementary File 1

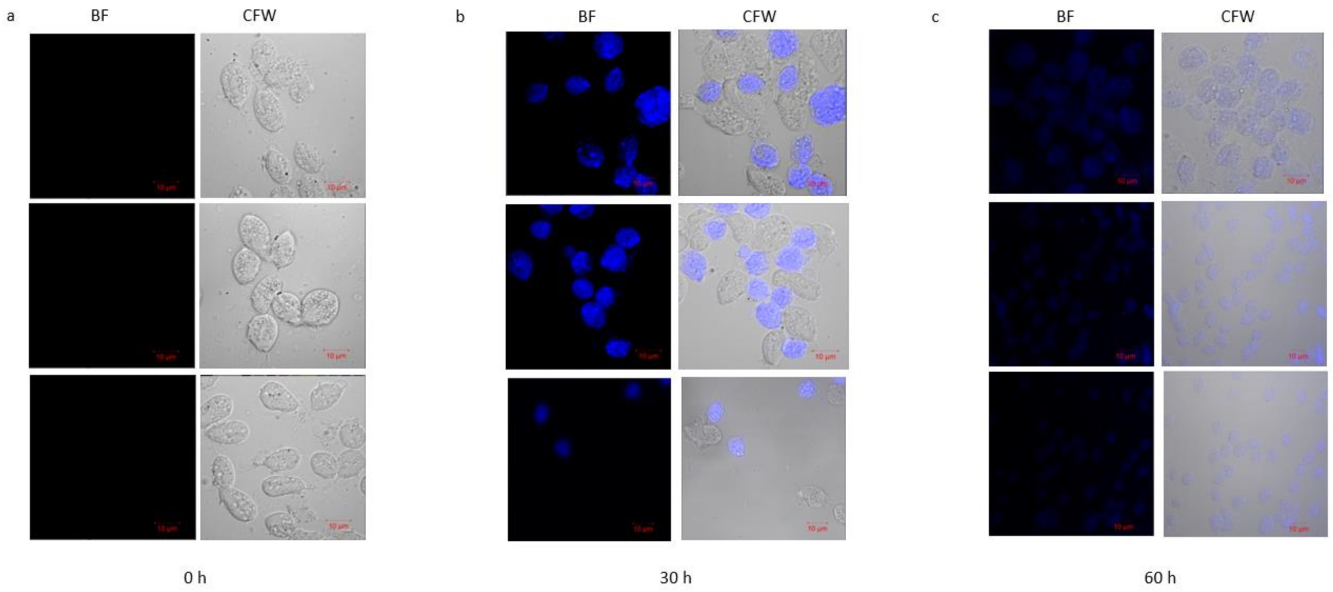

Supplementary Fig. 1: Encystation of trophozoites to cyst-like structures in *Trichomonas vaginalis.* (a) At 0h, all the parasites were in the motile trophozoite form. No cells were stained with CFW (b) After 30 hrs, some cells were observed to be spherical with internalized flagella and were stained with CFW. These are the cyst-like structures (CLS) of the parasite. (c) At 60 hrs, only spherical, non-motile CLS survived and were stained by CFW. CFW: Calcofluor White stain. *Scale bar: 10 µm.*

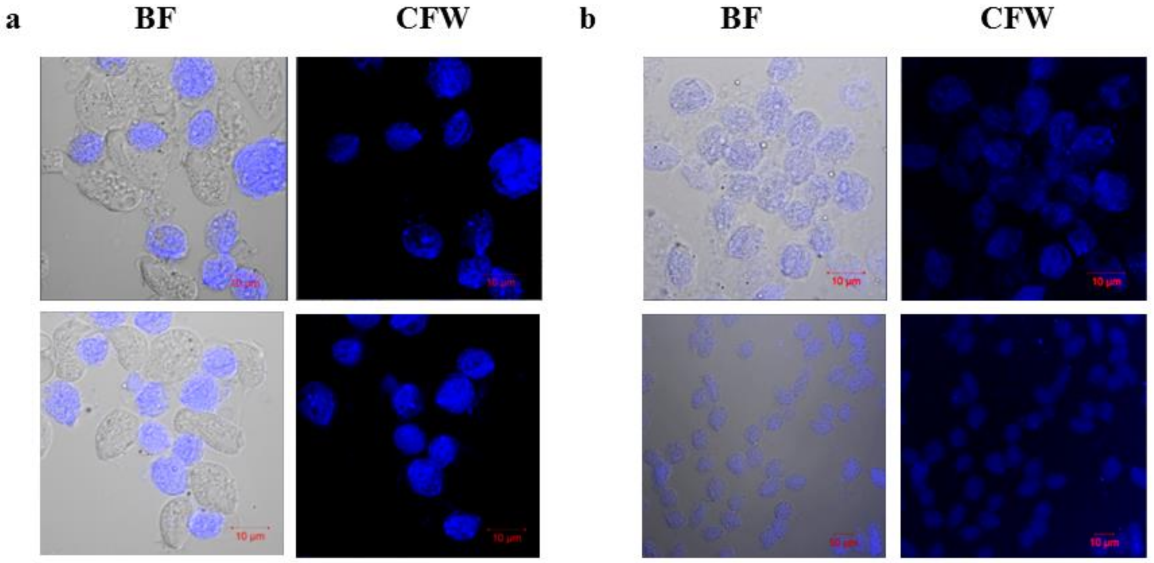

Supplementary Fig. 2: Enrichment of cyst-like structures of *T. vaginalis.* (a) As described under Methods, *T. vaginalis* cells were grown for 48 hrs and stained with CFW. About 60% cells fluoresced blue and were thus, described as CLS. (b) These cells were resuspended in sterile water and incubated for 12 hrs at 4°C. Post incubation, CFW staining was performed. All cells showed blue fluorescence and therefore were enriched *T. vaginalis* CLS. No trophozoites were seen. *Scale bar: 10 µm. (BF: Bright Field, CFW: Calcofluor White stained).*

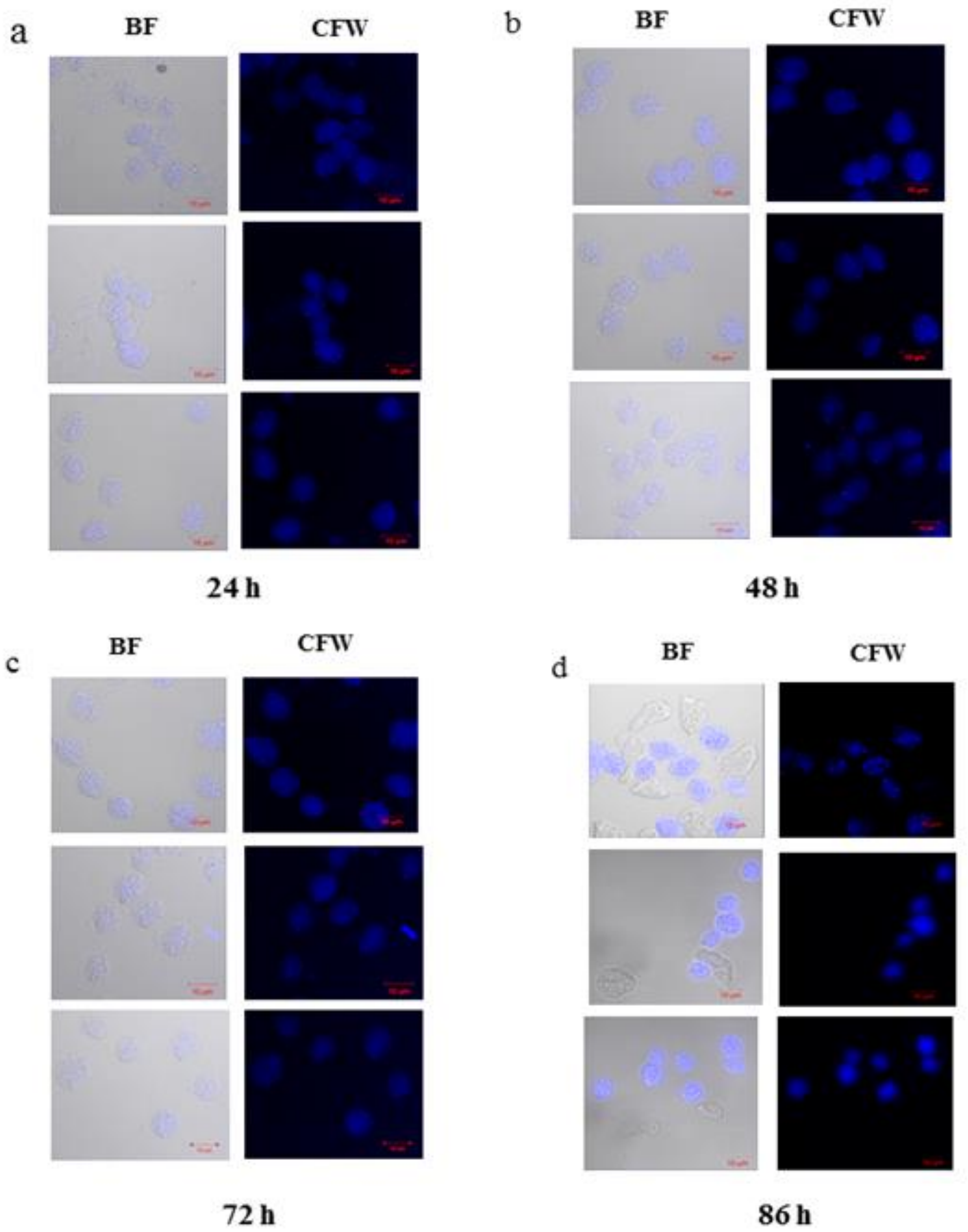

Supplementary Fig. 3: Excystation of cyst-like structures to trophozoites in *Trichomonas vaginalis*. Enriched CLS was transferred to fresh TYI medium and imaged at 24 ,48, 72 and 86 hrs. At 0 hrs, all parasites were stained by CFW (a) at 24 hrs, all cells were stained by CFW and thus, represented CLS exclusively. These were non-motile, and a few showed external flagella (b) At 48 hrs, most CLS were stained by CFW and had external flagella. (c) At 72 hrs, the cells showed prominent external flagella but still stained with CFW indicating presence of chitin containing CLS. (d) After 86 hours, some of cells became pear-shaped and motile. These cells did not stain with CFW and were motile; and thus, represented *T. vaginalis* trophozoites. *CFW: Calcofluor White stain. Scale bar: 10 µm.*

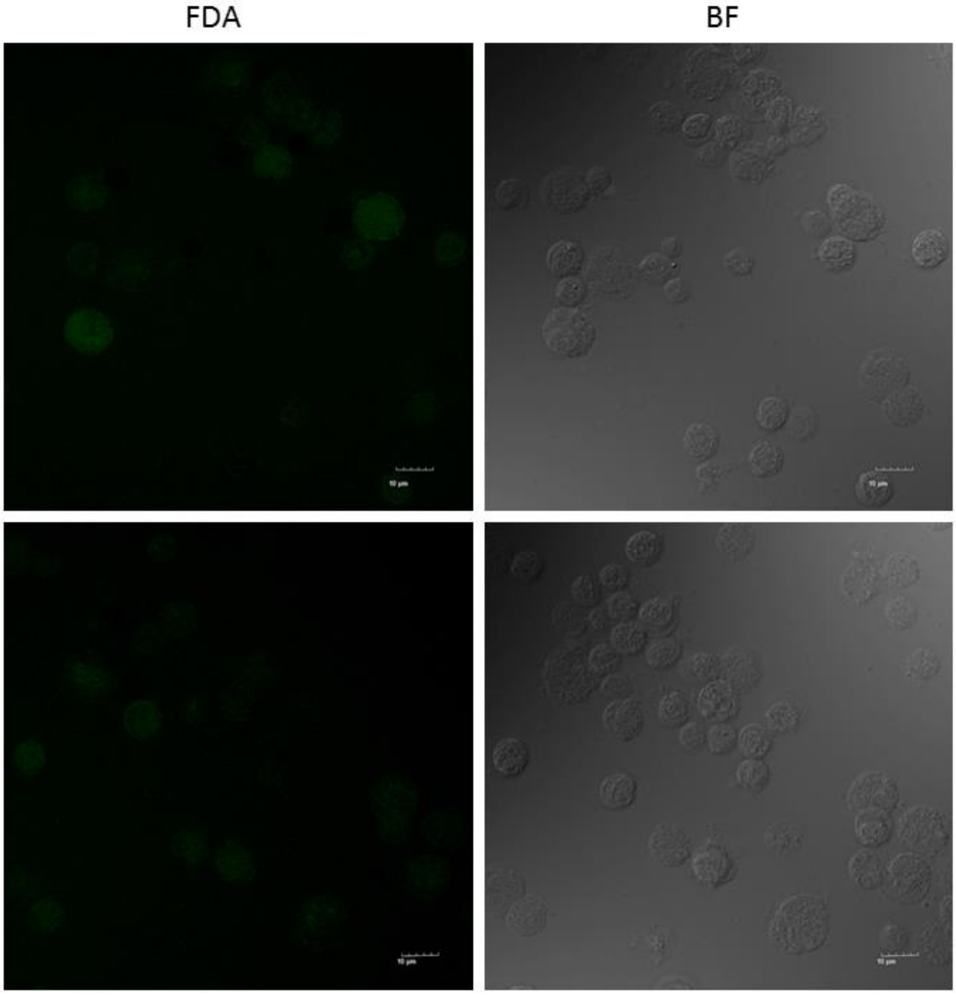

Supplementary Fig. 4: Viability of *T. vaginalis* cyst-like structures (CLS). (a) Enriched *T. vaginalis* cyst-like structures were incubated with Fluorescein Diacetate, a dye used to measure cell viability, as detailed under Methods. About 40% of these structures showed green fluorescence indicating viability of *T. vaginalis* cyst-like structures *FDA: Fluorescein Diacetate, BF: Bright field Scale Bar:10 µm.*

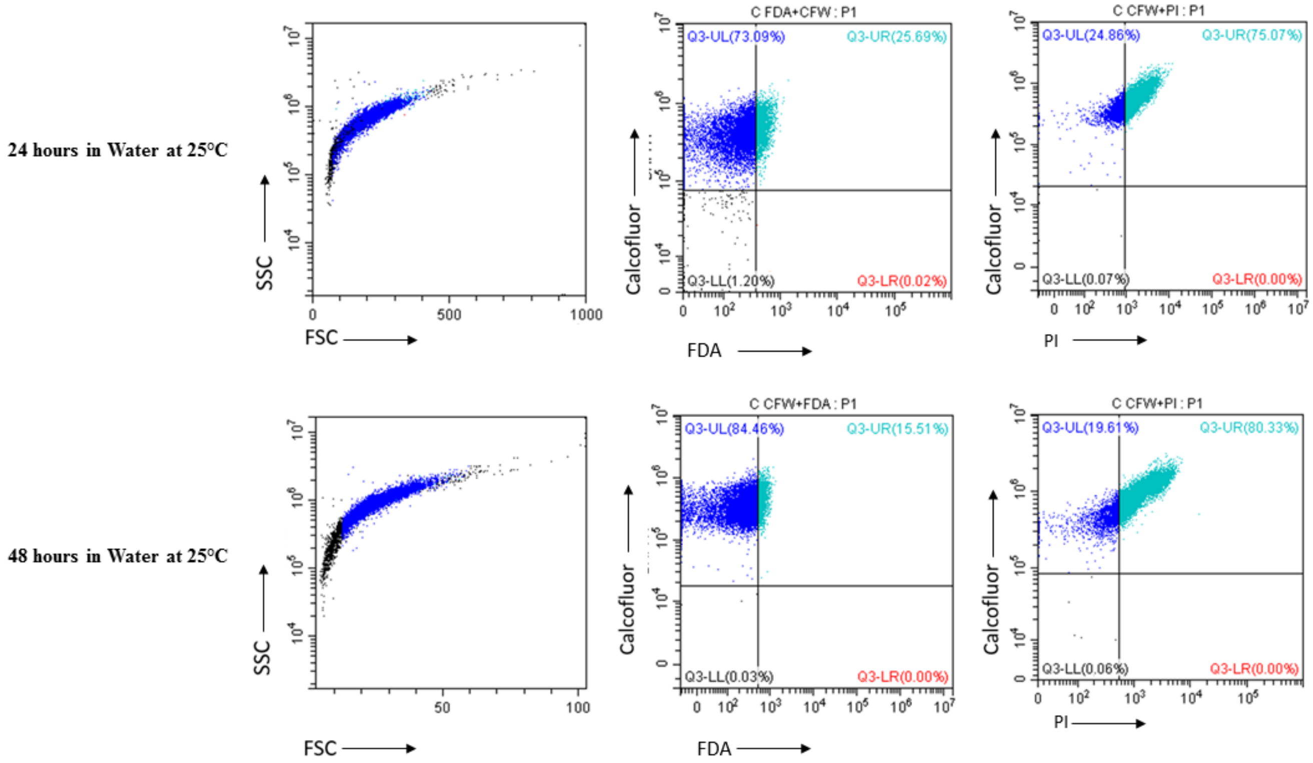

Supplementary Fig. 5: Viability of *T. vaginalis* CLS in water under prolonged periods of time. (a) Enriched *T. vaginalis* CLS (stains with CFW) was incubated in water at 25°C up to 48 hrs on end-to-end rotor. At 24 hrs and 48 hrs, viability was measured by flow cytometry using two dyes: FDA and PI staining. FDA provides a measure of viable cells while PI is indicative of dead cells. At 24 hrs of incubation in water, 25% of CFW-stained CLS were FDA positive and ~75% were PI positive while at 48 hrs, the viability measured by FDA staining reduced to ~15%. *FDA: Fluoroscein Diacetate, CFW: Calcofluor white, PI: Propidium Iodide.*

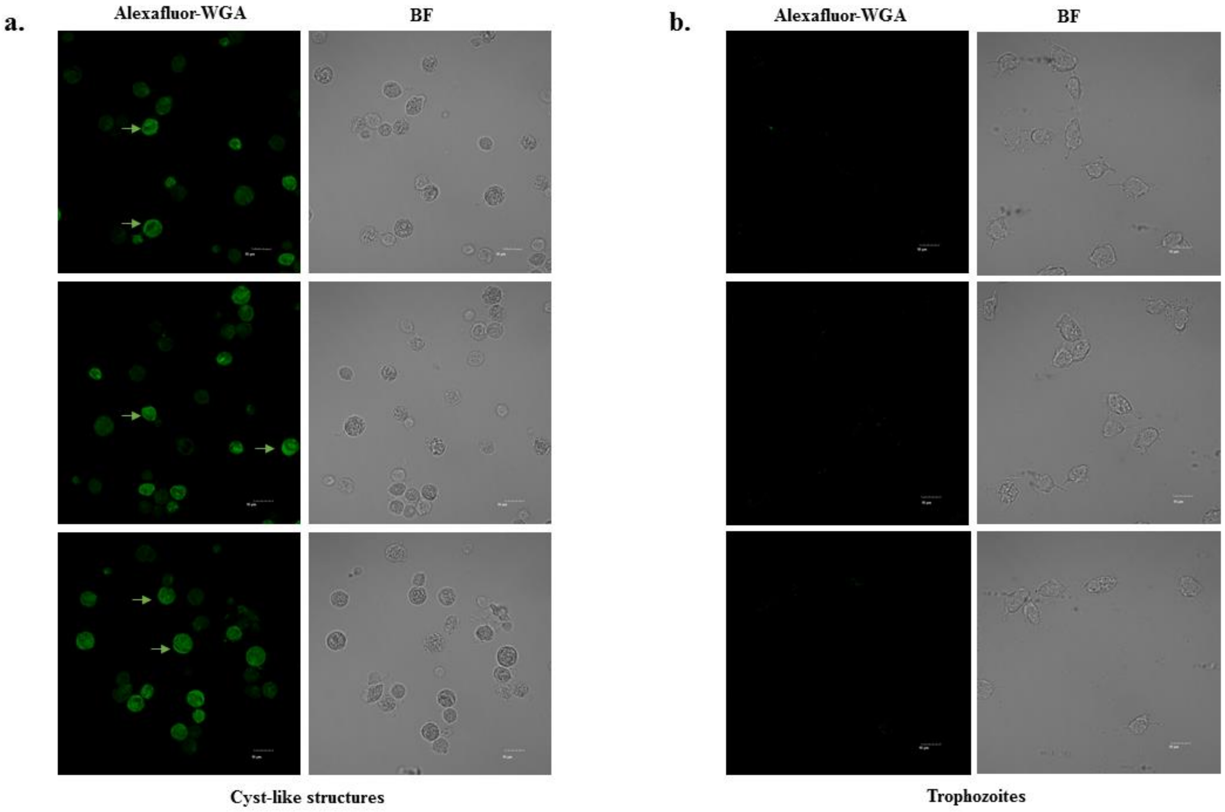

Supplementary Fig 6: Wheat-Germ Agglutinin-targeted fluorescence microscopy shows the presence of chitin in *T. vaginalis* CLS. WGA uniquely binds to N-acetyl glucosamine which is the building block of chitin. **(**a) Enriched CLS were stained with Alexafluor tagged WGA and viewed under a fluorescence microscope. CLS stained green, thus signifying that chitin is present in *T. vaginalis* CLS (b) Similarly, trophozoites were incubated with Alexafluor-WGA. Fluporescence microscopy clearly shows absence of binding of WGA to *T. vaginalis* trophozoutes; thus, signifying that this form is devoid of chitin. *BF: Bright Field, Alexafluor WGA: Alexafluor tagged to Wheat germ agglutinin. Scale Bar:10µm.*

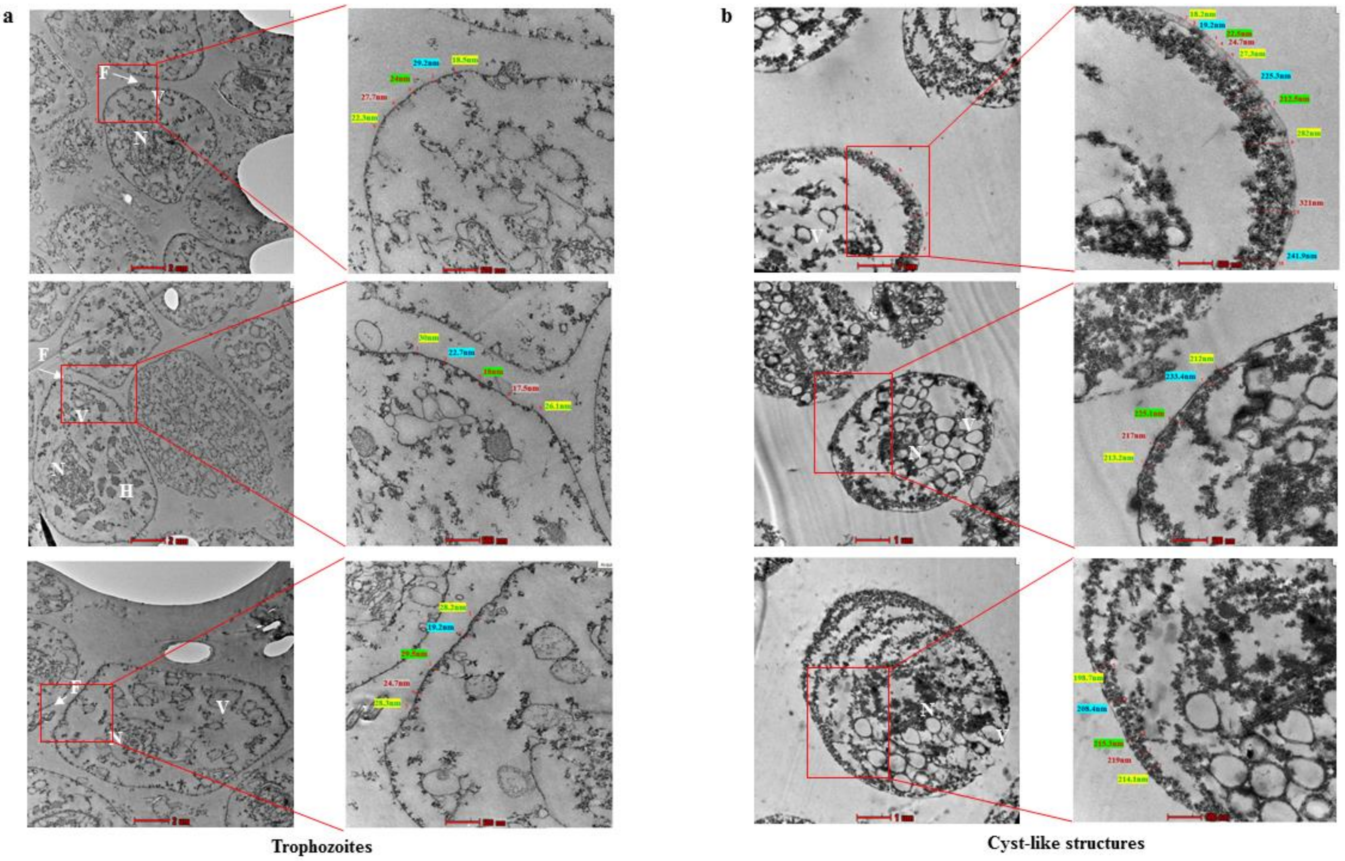

Supplementary Fig 7: Transmission Electron Microscope (TEM) image of *T. vaginalis*. (a) The images show flagellated, oval to elongated *T. vaginalis* trophozoites. Prominent structures have been marked. The characteristic shape of trophozoites is evident from the image. (b) The images show rounded, smaller *T. vaginalis* CLS with electron-dense deposition in their membrane. N: Nucleus, H: Hydrogenosome, V: Vacuole, F: Flagellum
