# Supplementary material for: Demonstration and Characterization of Cyst-Like Structures in the Life Cycle of Trichomonas vaginalis
Source: Front Cell Infect Microbiol. 2020 Jan 14;9:430. doi: 10.3389/fcimb.2019.00430 (PMC6972724; doi:10.3389/fcimb.2019.00430)
Supplement: Supplementary Methods — Liquid Chromatography and Mass spectrometry parameters for proteome analysis of T. vaginalis trophozoite and CLS. [file Data_Sheet_2.PDF]

Supplementary Methods

Liquid Chromatography Parameters

- Mobile Phase A: 0.1% FA in Water
- Mobile Phase B: 0.1% FA in Acetonitrile
- Flow rate: 0.400 mL/min
- Column: Agilent AdvanceBio Peptide Map 2.1*150mm, 2.7µ
- Injection volume: 20µL

Gradient Mobile Phase Composition

Time (min)	% A	% B
0	98	2
2	98	2
75	65	35
80	5	95
90	5	95
91	98	2
95	98	2

MS/MS Parameters

(Agilent 6545XT AdvanceBio LC/Q-TOF)

ESI Source parameters		MS/MS parameters	
Ionization source:	Electrospray ionization (Dual AJS ESI)	Acquisition mode:	Auto MS2
Ionization mode:	Positive	MS range (Precursor mass):	300-1700 m/z
Capillary voltage:	3.5 kV	MS scan rate (spectra/sec):	8
Gas Temp. (°C)	300	MS/MS range (Fragment mass):	50-1700 m/z
Gas Flow (L/min)	10	MS/MS scan rate (spectra/sec):	3
Nebulizer (psig)	35	Isolation width for MS/MS:	Narrow (~1.3 Da)
Sheath Gas Temp. (°C)	350	Max precursor per cycle	12
Sheath Gas Flow (L/min)	11	Collision energy	Ramped
